# Enrichment of inflammatory bowel disease and colorectal cancer risk variants in colon expression quantitative trait loci

**DOI:** 10.1186/s12864-015-1292-z

**Published:** 2015-02-27

**Authors:** Imge Hulur, Eric R Gamazon, Andrew D Skol, Rosa M Xicola, Xavier Llor, Kenan Onel, Nathan A Ellis, Sonia S Kupfer

**Affiliations:** Committee on Genetics, Genomics and Systems Biology, Chicago, IL 60637 USA; Department of Medicine, 900 East 57th Street, MB#9, Chicago, IL 60637 USA; Department of Pediatrics, University of Chicago, Chicago, IL 60637 USA; Department of Medicine, Yale University, New Haven, CT 06510 USA; University of Arizona Cancer Center, Tucson, AZ 85724 USA; Division of Genetic Medicine, Department of Medicine, Vanderbilt University, Nashville, TN 37232 USA

**Keywords:** Expression quantitative trait loci, Colon, Gene expression, African Americans, Regulatory variation, Transcriptomics, Inflammatory bowel disease, Colorectal cancer, Genomics, Genome-wide association studies

## Abstract

**Background:**

Genome-wide association studies (GWAS) have identified single nucleotide polymorphisms (SNPs) associated with diseases of the colon including inflammatory bowel diseases (IBD) and colorectal cancer (CRC). However, the functional role of many of these SNPs is largely unknown and tissue-specific resources are lacking. Expression quantitative trait loci (eQTL) mapping identifies target genes of disease-associated SNPs. This study provides a comprehensive eQTL map of distal colonic samples obtained from 40 healthy African Americans and demonstrates their relevance for GWAS of colonic diseases.

**Results:**

8.4 million imputed SNPs were tested for their associations with 16,252 expression probes representing 12,363 unique genes. 1,941 significant *cis-*eQTL, corresponding to 122 independent signals, were identified at a false discovery rate (FDR) of 0.01. Overall, among colon *cis-*eQTL, there was significant enrichment for GWAS variants for IBD (Crohn’s disease [CD] and ulcerative colitis [UC]) and CRC as well as type 2 diabetes and body mass index. *ERAP2*, *ADCY3, INPP5E, UBA7, SFMBT1, NXPE1* and *REXO2* were identified as target genes for IBD-associated variants. The CRC-associated eQTL rs3802842 was associated with the expression of *C11orf93* (*COLCA2*)*.* Enrichment of colon eQTL near transcription start sites and for active histone marks was demonstrated, and eQTL with high population differentiation were identified.

**Conclusions:**

Through the comprehensive study of eQTL in the human colon, this study identified novel target genes for IBD- and CRC-associated genetic variants. Moreover, bioinformatic characterization of colon eQTL provides a tissue-specific tool to improve understanding of biological differences in diseases between different ethnic groups.

**Electronic supplementary material:**

The online version of this article (doi:10.1186/s12864-015-1292-z) contains supplementary material, which is available to authorized users.

## Background

Genetic susceptibility is thought to play a role in common diseases including those affecting the colon such as inflammatory bowel diseases (IBD) and colorectal cancer (CRC). Indeed, genome-wide association studies (GWAS), conducted primarily in populations of European descent, have identified single nucleotide polymorphisms (SNPs) associated with IBD, including both ulcerative colitis (UC) [1-6] and Crohn’s disease (CD) [7-19], as well as CRC [20-30]. As is the case for GWAS variants in general, a number of these variants are located in gene deserts and their functional roles in disease pathogenesis are largely unknown [31]. Unraveling the functional basis of complex diseases is a priority as this has implications for understanding disease pathogenesis as well as identifying novel therapeutic targets [32].

Studying the genetics of gene expression is a tool that can help elucidate the functional consequences of GWAS variants. Expression quantitative trait loci (eQTL) mapping associates genome-wide SNPs with mRNA expression from the same individuals in a particular tissue to identify regulatory variation [33]. Previous studies [34-38] have detected eQTL in modest sample sizes (compared to typical disease GWAS)*.* These studies have provided important insights into the architecture of gene regulation in general [39], as well as across populations [40,41] and tissues [41-43]. Importantly, it has been shown that variants identified by GWAS as reproducibly associated with complex traits are enriched for eQTL in various cell types [33,44,45].

While eQTL mapping studies have been performed in lymphoblastoid cell lines (LCLs) [36,37,40,42,46], liver [35,47], adipose tissue [42], brain [48], skin [42,46] and ileum [49], these tissue types may not be relevant for all disease traits. A recent eQTL mapping study in the human ileum noted tissue-specific effects as well as enrichment for IBD susceptibility variants [49]. More recently, Closa *et al.* conducted an eQTL analysis of CRC loci in colonic mucosa and found significant *cis*-eQTL in three loci [50]. However, comprehensive genome-wide eQTL mapping has not previously been performed in the human colon, which is the relevant tissue for colonic diseases like IBD and CRC.

The goal of the present study was to comprehensively map eQTL in healthy human colon in order to characterize colon-specific gene regulation and evaluate its relevance for GWAS of IBD and CRC as well as other complex phenotypes. Towards this end, genome-wide genotyping, using a microarray optimized for individuals of African descent, and gene expression profiling were performed colonic tissue obtained from 40 African American (AA) healthy subjects who underwent screening colonoscopies. These findings and resources will allow for improved understanding of disease pathogenesis of inflammatory and malignant diseases of the colon.

## Results

In order to identify significant colon eQTL, the genotype and expression data was first subjected to a number of quality controls. Using the cleaned data, *cis-* and *trans-*eQTL were identified. The overlap between colon eQTL and disease-associated GWAS SNPs was performed as well as the overlap with eQTL in other tissue types. As only AA were used in this study, population differentiated eQTL were also identified. Finally, using simulations, enrichment of colon eQTL among IBD- and CRC-associated variants and for histone marks was performed. Results for these analyses are presented in this order in the sections that follow.

### Quality control of genotype and expression data

**Figure 1:**
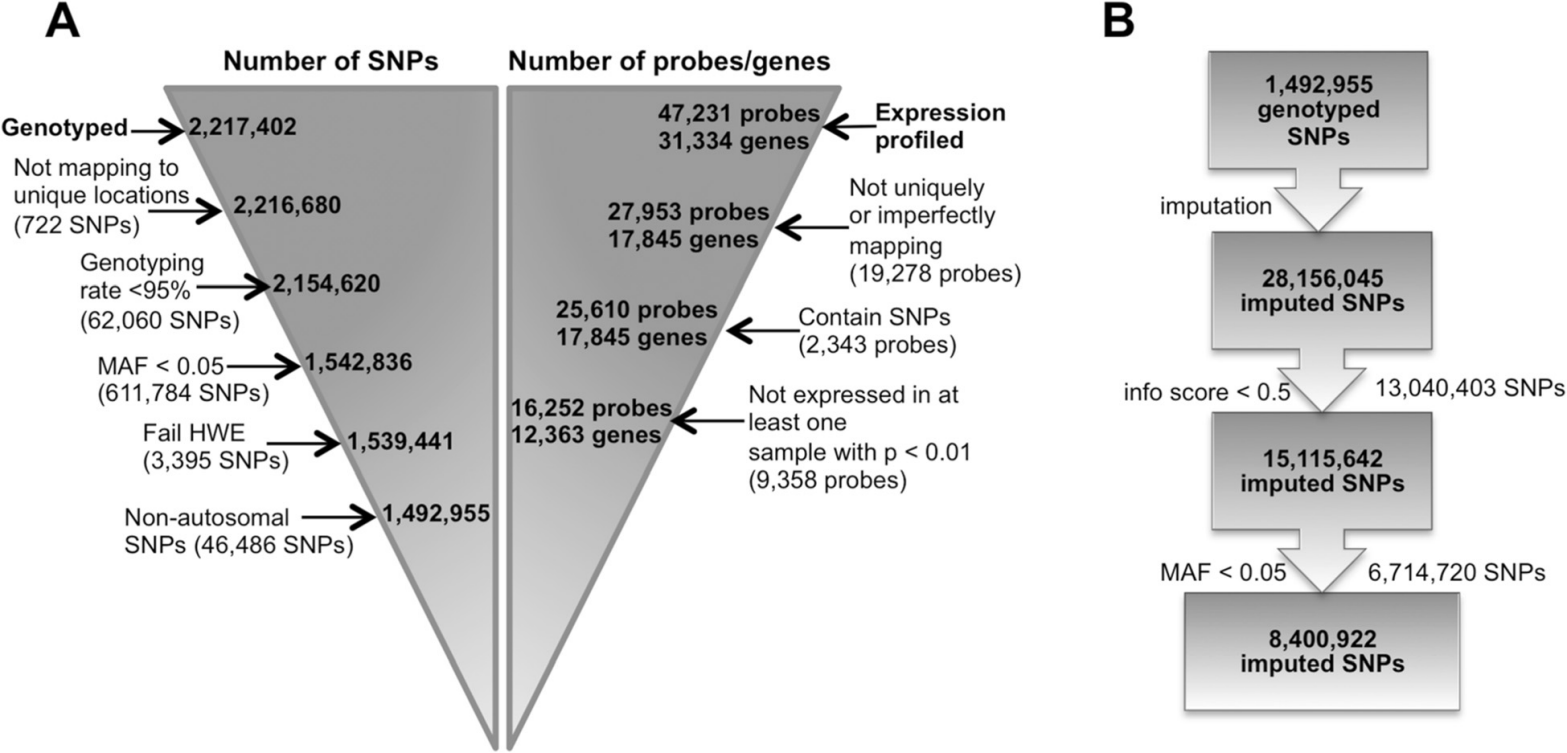
**Flowchart summarizing the study design. (A)** Flowchart describing the quality control (QC) process for the SNP genotype and gene expression data (See Methods for details). The numbers inside the triangles correspond to the numbers of SNPs or probes/genes that are left after the removal of those (numbers given inside parentheses) that fail to meet the QC criteria outlined in the text next to the arrows. 1,492,955 genotyped SNPs passed the QC filters and were imputed. 16,252 gene expression probes corresponding to 12,363 unique genes passed the QC filters and were included in the eQTL analysis. **(B)** Flowchart summarizing the imputation and post-imputation QC steps for the 1,492,955 genotyped SNPs that passed QC in **(A)** and were imputed using 1000 Genomes as reference to provide data on 28,156,045 SNPs (see Methods for details). The description of each step along with the numbers of SNPs that were excluded at each is listed next to the arrows. The final dataset consisted of 8,400,922 imputed SNPs in 48 individuals.

2.2 million SNPs were genotyped in 48 AA subjects using the Affymetrix Axiom Pan-African array (Figure 1A). SNPs were removed if they had a genotyping rate <95% (n = 62,060), minor allele frequency (MAF) < 0.05 (n = 611,784), significant Hardy-Weinberg equilibrium (HWE) p-values (n = 3,395, see Methods), non-autosomal SNPs (n = 46,486), or mapped to the same position (n = 722) as another SNP. Imputation was performed using 1.492 million remaining SNPs and the 1000 Genomes Project reference panel Phase I integrated variant set release (v3), which provided a final dataset of 8.4 million SNPs, after removing imputed SNPs with MAF < 0.05 or low imputation quality (IMPUTE2-info score < 0.5) (Figure 1B). Principal components analysis (PCA) showed that subjects included in this study were similar to individuals of African ancestry in Southwest USA (ASW) HapMap population samples in terms of ancestry (Additional file 1). The proportion of European and African ancestry among the subjects were represented by the first principal component (PC); PC1 was thus used as a covariate in the eQTL analyses to control for global ancestry.

In gene expression analysis, 47,231 probes were profiled. A total of 16,252 probes remained for analysis after removing probes that mapped to more than one genomic location (n = 19,278), that contained one or more SNP (n = 2,343), or that was not expressed in one or more subjects (n = 9,358). Heat map visualization and hierarchical clustering of the gene expression data suggested the removal of eight outliers, leaving 40 individuals (Additional file 2). Inclusion of the first five PCs of the gene expression data yielded the maximal number of significant *cis*-eQTL probes at various false discovery rate (FDR) thresholds (Additional file 3). These expression-based PCs were included as covariates in subsequent eQTL analysis to correct for unmeasured yet systematic variation in gene expression levels.

### Identification of colon eQTL

eQTL were identified by testing for an association between each SNP and each gene’s expression probe. The distribution of eQTL p-values was compared against the distribution expected by chance separately for *cis-* and *trans-*eQTL. For *cis*-, but not *trans*-eQTL, we found a significant enrichment for small p-values (Additional file 4). We identified 1,941 *cis*-eQTL corresponding to 122 genes at a FDR of 0.01, which represent 122 independent SNP-gene associations (Table 1). *Cis*-eQTL were found to be highly enriched around transcription start sites (TSS) with no discernible trend toward 3′ or 5′ (Additional file 5).

**Table 1:** **Numbers of significant colon** ***cis*** **-eQTL identified at four FDR thresholds**

	**Number of** ***cis*** **-eQTL**			
**FDR**	**SNP-gene pairs**	**SNPs**	**Independent** ^*^	**Genes**
0.01	1,941	1,941	122	122
0.05	5,183	5,181	353	352
0.10	8,252	8,122	693	684
0.20	14,255	14,177	1,677	1,614

*These numbers correspond to the numbers of independent SNP-gene associations that were determined using the stepwise association method outlined in Supplementary Methods (Additional file 8).

### Overlap of *cis*-eQTL with colonic disease-associated GWAS variants

**Figure 2:**
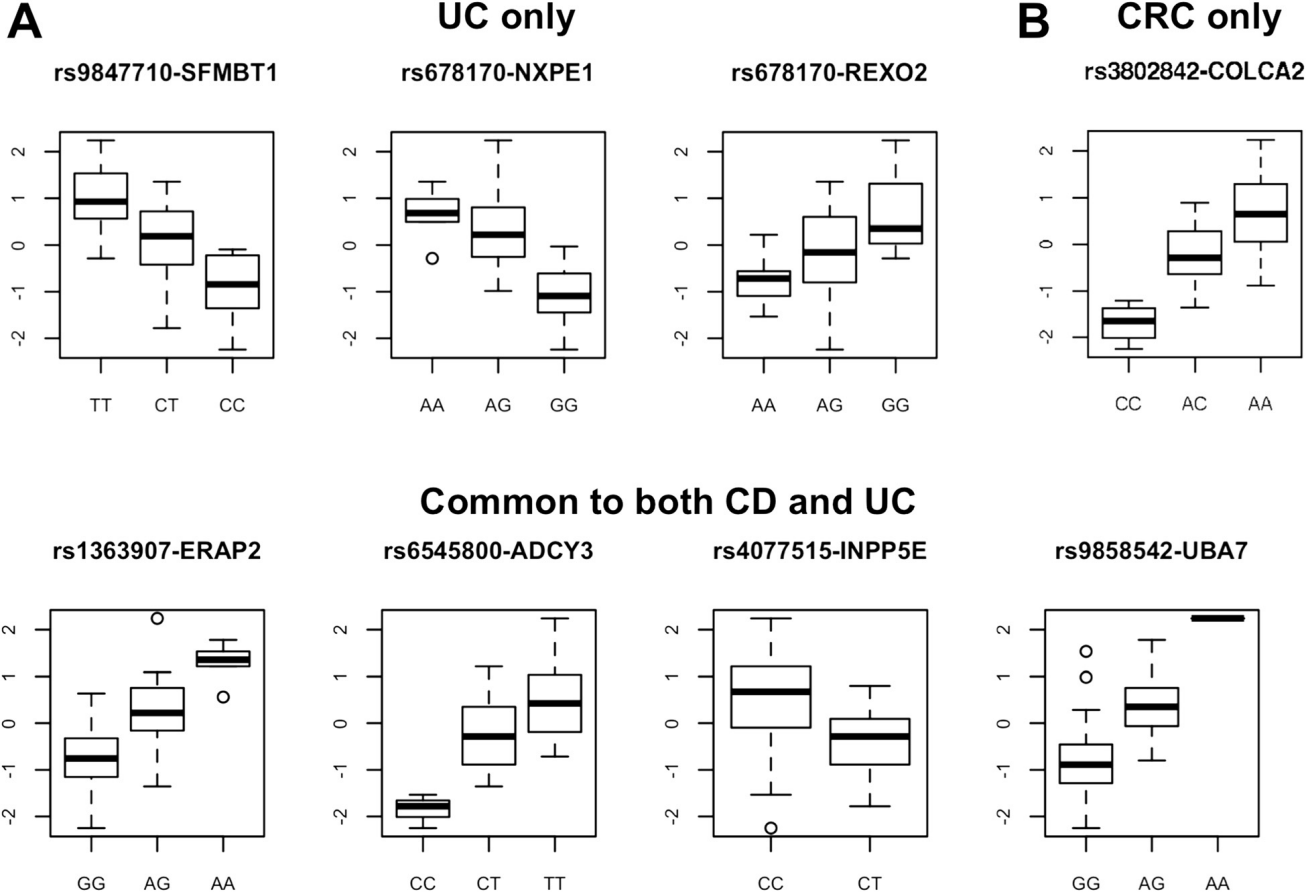
**Colon** ***cis-*** **eQTL that are also associated with colonic diseases.** The box plots depict the relationship between SNPs associated with **(A)** IBD (i.e. CD and/or UC) or **(B)** CRC from the NHGRI GWAS catalog and their target gene’s expression. The x-axes correspond to the SNP genotypes, and the y-axes represent the log2-normalized gene expression values. The median gene expression level for each genotype is indicated by a horizontal line with the boxes covering 25th and 75th percentiles and the whiskers extending to 1.5 times the interquartile range. Points outside the whiskers are plotted as outliers. For each target gene, the disease-associated SNP was selected for the box plot even if it is not the most significant *cis*-eQTL (but must be in r^2^ ≥ 0.8 with it).

The National Human Genome Research Institute (NHGRI) GWAS catalog was searched to identify IBD (i.e. CD and/or UC)- and CRC-associated GWAS variants that are also significant *cis*-eQTL at FDR < 0.10 in the colon samples. Of 8,122 significant *cis*-eQTL at this threshold, 4 were previously identified in GWAS of IBD (i.e. both CD and UC), CD only, UC only or CRC. When SNPs in high linkage disequilibrium (LD) (r^2^ ≥ 0.8) with the GWAS variants were included, the overlap between GWAS signals and significant *cis-*eQTL increased to 127 variants: 103 common to CD and UC, 6 for UC-only and 18 for CRC. These significant *cis*-eQTL corresponded to 8 unique target genes that co-localize with disease-associated SNPs (Figure 2; Table 2).

**Table 2:** **Colon** ***cis*** **-eQTL that are associated with Crohn’s disease (CD) and/or ulcerative colitis (UC) as well as colorectal cancer (CRC)**

	**SNP/gene**	Proxy SNP* (r ^**2**^ **)**	**eQTL p-value**
**Both CD and UC**	rs1363907/*ERAP2*		1.4 × 10^−8^
	rs6545800/*ADCY3*	rs2384061 (r^2^ = 0.82)	1.3 × 10^−7^
	rs4077515/*INPP5E*	rs4266763 (r^2^ = 0.95)	4.7 × 10^−6^
	rs9858542/*UBA7*	rs10640 (r^2^ = 0.92)	8.7 × 10^−6^
**CD only**	rs2549794/*ERAP2*		1.5 × 10^−8^
**UC only**	rs9847710/*SFMBT1*		8.3 × 10^−6^
	rs6781710/*NXPE1 & REX02*	rs661946 (r^2^ = 0.94)	8.7 × 10^−7^
**CRC**	rs3802842/*COLCA2*		1.1 × 10^−6^

*SNPs in LD (r^2^ ≥ 0.8) with disease-associated GWAS SNPs from the NHGRI catalog. r^2^ corresponds to the correlation between the proxy SNP and the GWAS SNP, calculated based on the 48 African American samples in the colon dataset.

### Comparison of colon *cis*-eQTL with other tissues

A comparative analysis was performed to assess the extent of overlap between colon *cis*-eQTL and *cis*-eQTL for other tissue types (Table 3). Substantial percentages of colon *cis*-eQTL target genes were found to be *cis*-eQTL target genes in other tissues, including genes in LCLs, liver, brain, skin, and ileum. As the FDR threshold for defining *cis*-eQTL in the colon became more stringent, the percentage overlap of colon *cis*-eQTL target genes with *cis*-eQTL target genes from other tissues increased.

**Table 3:** **Comparison of colon** ***cis*** **-eQTL results with published studies in different tissues**

			**FDR**			
	**Samples**		**0.01**	**0.05**	**0.10**	**0.20**
**LCL** [41]	13,643 genes and ~2.2 million SNPs in 270 HapMap subjects (CEU, CHB, JPT and YRI populations)	Genes*	25 (20%)	49 (14%)	72 (11%)	119 (7.4%)
		SNP-gene pairs**	130	182	298	361
**Liver** [35]	34,266 genes and 782,476 SNPs in liver samples acquired from 427 Caucasian subjects	Genes*	41 (34%)	95 (27%)	169 (25%)	346 (21%)
		SNP-gene pairs**	8	15	20	33
**Brain** [48]	22,184 genes and 1,629,853 SNPs in brain tissue samples from 150 Caucasian subjects	Genes*	17 (14%)	35 (9.9%)	63 (9.2%)	137 (8.5%)
		SNP-gene pairs**	139	191	277	327
**Skin** [46]	14,500 genes and 438,670 SNPs in skin samples from 57 Caucasian subjects	Genes*	24 (20%)	39 (11%)	59 (8.6%)	92 (5.7%)
		SNP-gene pairs**	131	92	71	50
**Ileum** [49]	19,047 genes and 581,633 SNPs in ileum samples from 173 mostly Caucasian subjects	Genes*	37 (30%)	83 (24%)	118 (17%)	197 (12%)
		SNP-gene pairs**	176	130	94	58

*The number of target genes from other studies overlapping colon *cis*-eQTL target genes are listed along with the percentages of overlap (in parentheses). To calculate the percentage of overlap, the number of overlapping target genes was divided by the total number of unique significant colon *cis*-eQTL target genes at the corresponding FDR threshold (refer to Table 1 for the actual numbers). **The number of SNP-gene pairs that overlap between the colon and other tissues.

### Population differentiated colon *cis*-eQTL

**Figure 3:**
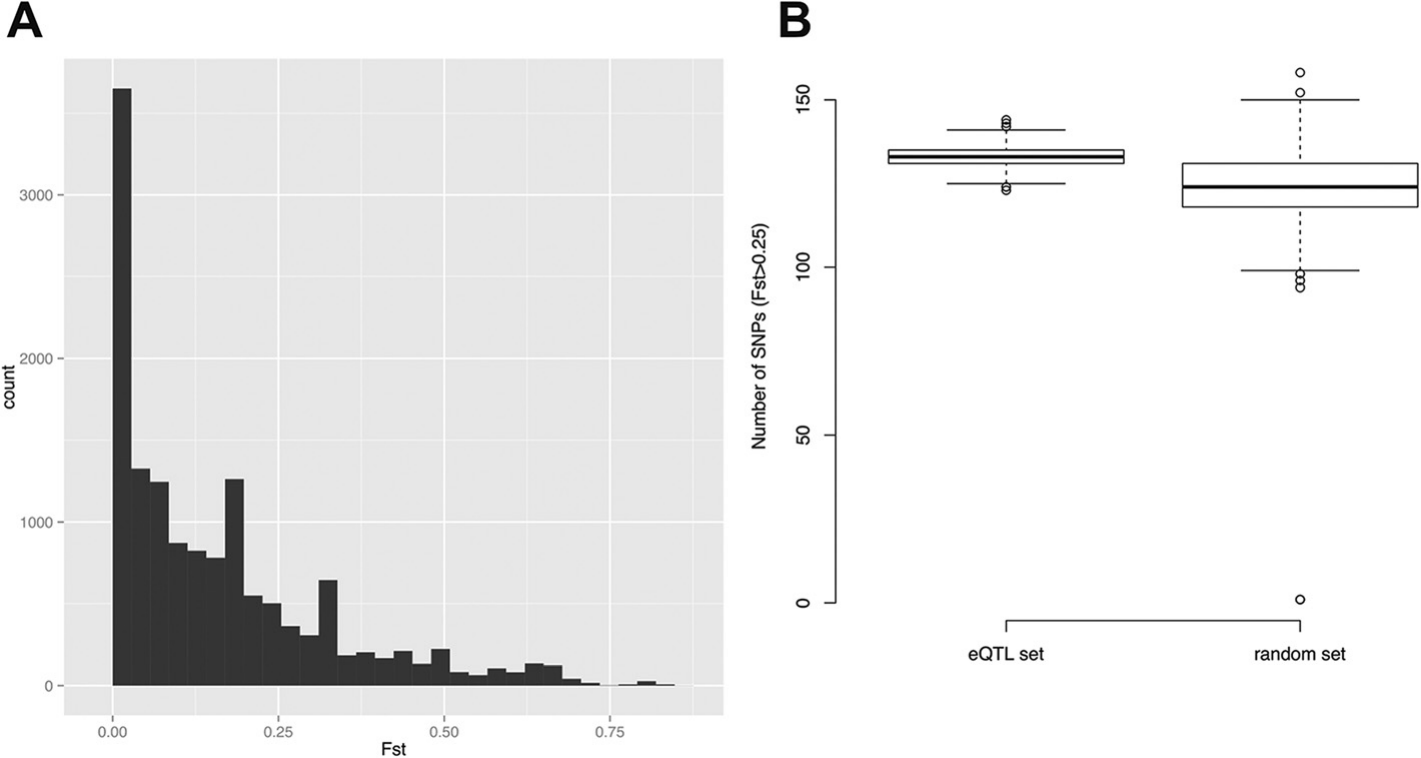
***Cis-*** **eQTL are enriched for SNPs that are highly differentiated between European and African populations.** F_ST_ values for the study SNPs were calculated between 1000 Genomes Project European (EUR) and African (AFR) populations using Weir and Cockerham’s unbiased estimator. SNPs with F_ST_ > 0.25 were defined as population differentiated SNPs. **(A)** The histogram shows the distribution of F_ST_ values for the significant colon *cis*-eQTL (FDR < 0.20). Among the 14,135 *cis*-eQTL for which F_ST_ estimates were obtained, 3,185 (23%) were population differentiated. **(B)** Enrichment of population differentiated SNPs among significant colon *cis*-eQTL was evaluated using a simulation-based method. The box plot depicts the distributions of the number of population differentiated SNPs among 1,000 randomly selected *cis*-eQTL SNP sets (left)―generated by randomly selecting a single SNP for each unique *cis*-eQTL target gene (n = 684) among all *cis*-eQTL (FDR < 0.20) that are significantly associated with the expression of that gene―and among 1000 random sets of SNPs (right), each matching the set of 684 significant *cis*-eQTL SNPs, based on the distributions of MAF and distance from the nearest TSS. The numbers of population differentiated SNPs among the eQTL and random SNP sets are indicated by horizontal lines with the boxes covering 25th and 75th percentiles and the whiskers extending to 1.5 times the interquartile range. The numbers of population differentiated SNPs in the eQTL sets were significantly higher than in the random sets of SNPs (p *<* 0.001 by Mann–Whitney test).

Fixation index (F_ST_) values were calculated between 1000 Genomes Project European (EUR) and African (AFR) populations for all significant colon *cis*-eQTL (FDR < 0.20). Out of the 14,177 significant *cis*-eQTL SNPs, F_ST_ estimates were successfully calculated for 14,135 SNPs. 3,185 *cis*-eQTL (23% of all *cis*-eQTL for which F_ST_ values were estimated) had F_ST_ > 0.25, indicating high population differentiation (Figure 3A) which was greater than that expected under the null at all FDR thresholds tested (0.2, 0.1, 0.05, 0.01; results at FDR < 0.20 in Figure 3B). Population differentiated SNPs that are also associated with CD, UC and CRC were identified among colon *cis*-eQTL using the F_ST_ statistic. As the mean value of F_ST_ between CEU and YRI has been estimated to be 0.071 across 1000 Genomes Project SNPs [51], F_ST_ threshold of 0.10 was used to define SNPs as population differentiated. For UC, there was one disease-associated SNP rs9847710 with F_ST_ > 0.10 (Additional file 6). For CD- and CRC-associated SNPs, there were no GWAS variants with F_ST_ > 0.10.

### Enrichment of colon *cis*-eQTL among IBD- and CRC-associated variants

**Figure 4:**
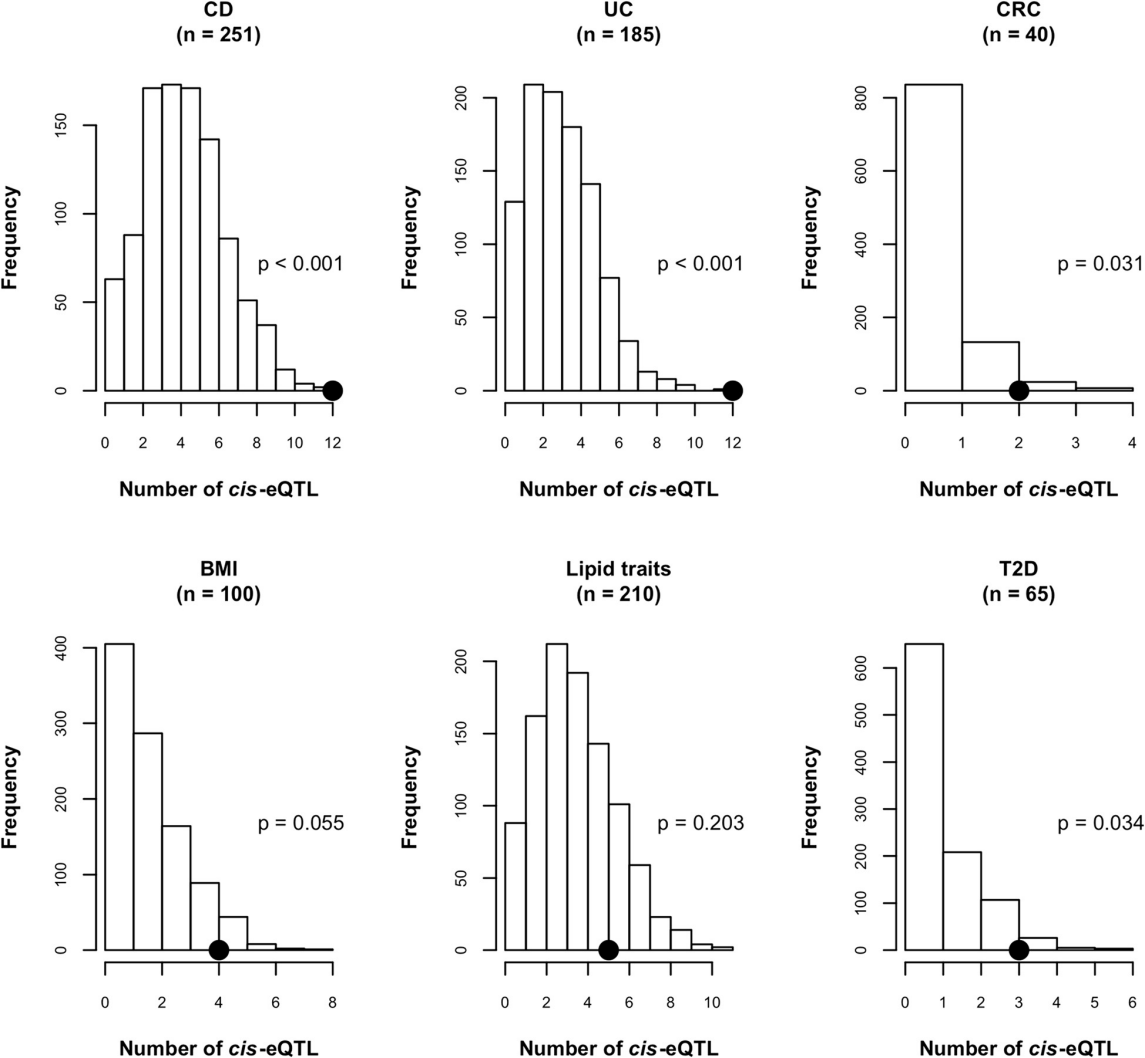
**SNPs associated with colonic diseases and type 2 diabetes are enriched for colon** ***cis*** **-eQTL.** A simulation-based analysis was performed to test for the enrichment of colon *cis*-eQTL among SNPs associated with colonic diseases **(A)** body mass index (BMI), lipid traits and type 2 diabetes (T2D) **(B)** downloaded from the NHGRI GWAS catalog. The distribution of the number of *cis*-eQTL in 1,000 simulated SNP sets, each of the same size (n) as the list of trait-associated SNPs and containing SNPs matched on MAF distribution is shown in the histograms. Solid black circles represent the actual *cis*-eQTL count (*cis*-eQTL p-value threshold of 0.001) observed in the trait-associated SNPs. The p-values shown are empirical, and are calculated as the proportion of sampled SNP sets in which the *cis*-eQTL count exceeds the actual count observed in the trait-associated SNPs. Enrichment of *cis*-eQTL among disease-associated SNPs is statistically significant for all colonic diseases. Enrichment of *cis*-eQTL is statistically significant for T2D (p = 0.034) and suggestively significant for BMI (p *=* 0.055). There is no enrichment of *cis*-eQTL among SNPs associated with lipid traits.

Simulation-based analysis was performed to investigate whether IBD- and CRC- associated variants are enriched for the 150,213 unique *cis*-eQTL SNPs with p *<* 0.001 compared with SNPs matched on MAF (see Methods for simulation details). Significant enrichment of *cis*-eQTL was observed among SNPs associated with CD (n = 251) and UC (n = 185) from the GWAS catalog, with n corresponding to the number of disease-associated SNPs included in the enrichment analysis, with empirical p-values for enrichment of < 0.001 (Figure 4A; Table 4). *Cis*-eQTL counts among the CRC-associated SNPs (n = 40) were also significantly higher than expected based on MAF distribution (p = 0.031).

**Table 4:** **Enrichment of colon** ***cis*** **-eQTL among NHGRI GWAS SNPs**

**Disease or trait**	**Number of GWAS SNPs**	**Number of GWAS SNPs that are colon** ***cis*** **-eQTL**	p-value*
**CD**	251	12	<0.001
**UC**	185	12	<0.001
**CRC**	40	2	0.031
**T2D**	65	3	0.034
**BMI**	100	4	0.055

*P-values were empirically determined using a simulation-based method in which 1,000 randomized SNP sets, matched in size and MAF distribution to the NHGRI GWAS SNPs, were generated. These simulations yielded a p-value, calculated as the proportion of sampled SNP sets in which the *cis*-eQTL count exceeds the actual count observed in the GWAS SNPs. For details see Supplementary Methods (Additional file 8).

In order to determine whether enrichment of colon eQTL is specific to particular complex traits or diseases, enrichment analysis was performed for SNPs associated with autoimmune disorders [celiac disease, psoriasis, rheumatoid arthritis (RA)], cancers (breast cancer, prostate cancer and melanoma), lipid traits (total cholesterol and triglyceride levels), body mass index (BMI), type 2 diabetes (T2D), and psychiatric disorders [attention deficit hyperactivity disorder (ADHD), bipolar disorder (BD) and schizophrenia]. There was statistically significant colon *cis-*eQTL enrichment among SNPs associated with T2D (p *=* 0.034) and suggestive enrichment among SNPs associated with BMI (p *=* 0.055) (Figure 4B; Table 4). SNPs associated with the other traits did not show enrichment for colon eQTL (Additional file 7; Additional file 8: Table S1).

### Enrichment of histone marks in colon *cis*-eQTL

**Figure 5:**
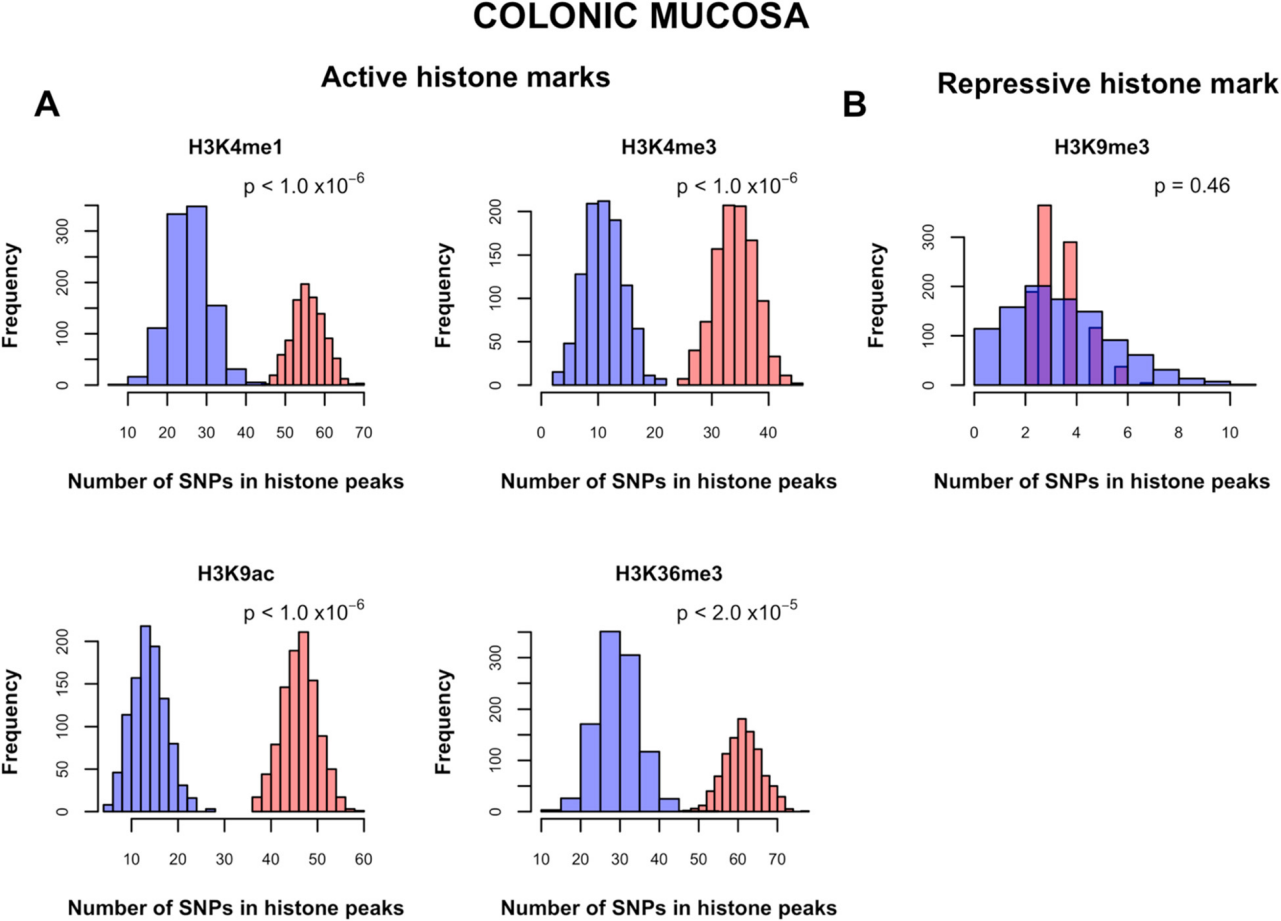
***Cis*** **-eQTL are enriched for active but not repressive histone marks in colonic mucosa.** The red histogram in each plot depicts the distribution of the number of SNPs in histone mark peaks in 1,000 randomly selected *cis*-eQTL SNP sets, which are generated by randomly selecting a single SNP for each unique *cis*-eQTL target gene (n = 684) among all *cis*-eQTL (FDR < 0.10) that are significantly associated with the expression of that gene. The blue histograms represent the distributions of the number of SNPs in histone mark peaks in 1,000 randomly sampled SNP sets, each matching the set of 684 significant *cis*-eQTL SNPs (chosen at random from the set of 1,000 *cis*-eQTL SNPs depicted in red) with respect to MAF and distance from the nearest TSS. Four markers of active chromatin (H3K4me1, H3K4me3, H3K9ac and H3K36me3) are depicted in **(A)**, while a single marker of inactive chromatin (H3K9me3) is depicted in **(B)**. The p-value in the top right corner of each histogram is the empirical p-value obtained by comparing the number of SNPs in histone mark peaks in the 1,000 sets of *cis*-eQTL SNPs (red) to the null distribution given by the 1,000 sets of matched SNPs (blue).

Simulations were conducted to test whether SNPs overlapping with activating (H3K4me1, H3K4me3, H3K9ac and H3K36me3) and repressing (H3K9me3 and H3K27me3) histone mark peaks were statistically enriched among *cis*-eQTL at an FDR of 0.10 (see Methods). Significant enrichment of active histone marks was noted among significant *cis*-eQTL with the following empirical p-values: < 1.0 × 10^−6^ (H3K4me1, H3K4me3 and H3K9ac) and 2.0 × 10^−5^ (H3K36me3) (Figure 5A). There was no significant enrichment for the repressive histone mark H3K9me3 among *cis*-eQTL (p = 0.46) (Figure 5B). A similar pattern of significant enrichment of active histone marks but not repressive histone marks among colon *cis*-eQTL was detected in colonic smooth muscle, adipose nuclei, adult liver, and breast myoepithelial cells (data not shown). In human CRC adenocarcinoma cell line (Caco-2), there was enrichment for H3K4me3 (p *<* 1.0 x 10^−6^) and a suggestive enrichment for H3K36me3 (p *=* 0.060), but no significant enrichment for the repressing mark H3K27me3 (p *=* 0.86) (Additional file 9). Taken together, these analyses show that colon *cis-*eQTL are enriched for active histone marks in several tissue types including malignant CRC cell lines.

## Discussion

Gene expression in a disease-relevant tissue is a powerful intermediate phenotype that could help elucidate the functional basis of some risk-associated variants identified in GWAS. Gene expression analysis is especially important as disease-associated variants are enriched in non-coding regions [33,44,52]. The analysis of published GWAS variants associated with colonic diseases noted significant enrichment for *cis-*eQTL in IBD (CD and UC) as well as CRC. Novel target genes for IBD-associated variants were identified and several previously reported eQTL identified in other tissues were validated. Moreover, overlap of colon eQTL in other tissue types, most notably ileum and liver was noted. Colon *cis-*eQTL were located near TSS as well as enriched for active histone marks and variants with high population differentiation, results that underscore the functional role of eQTL in gene regulation. This analysis could provide insights into the functional consequences of disease-associated genetic variants.

Among IBD-associated GWAS SNPs, variants were associated with differences in the expression of endoplasmic reticulum aminopeptidase 2 (*ERAP2*) and scm-like with four MBT domains 1 (*SFMBT1*) (Figure 2A; Table 2), similar to results in an eQTL mapping study of the human ileum [49]. *ERAP2* encodes an endoplasmic reticulum aminopeptidase responsible for major histocompatibility complex class I (MHC1) ligand trimming [53] that has *in silico* support as a functional variant in CD [10]. *SFMBT1* is a polycomb protein with transcriptional repressor activity [54,55] that may regulate a number of genes through epigenetic mechanisms. Additional SNPs for IBD-associated variants identified in this study and their gene targets*—*rs6545800 with adenylate cyclase (*ADCY3*), rs4077515 with inositol polyphosphate 5-phosphatase (*INPP5E*), rs9858542 with ubiquitin-like modifier activating enzyme 7 (*UBA7*)*,* and rs678170 with neuroexophilin and PC-esterase domain family member 1 (*NXPE1*) and with RNA exonuclease 2 (*REXO2*) *—*were novel targets and should be validated in a larger study.

Among CRC-associated GWAS SNPs, rs3802842, was found to be a *cis-*eQTL in the colon [26,56], a finding that was also recently reported by another study [50]. The target transcript *C11orf93* corresponds to an uncharacterized gene known as colorectal cancer associated 2 (*COLCA2*). A study found that two functional risk variants (rs7130173 and rs10891246), which are in perfect LD with rs3802842, lead to decreased expression of *C11orf93* [57]. These results were similar to our finding that the C allele of rs3802842 also results in decreased expression. While this variant was first identified in individuals of European descent, a previous study by our group validated this SNP as associated with rectal cancer in AA [58], though other groups have not found evidence for an association of rs3802842 with CRC in AA [59]. A recent trans-ethnic GWAS identified an additional SNP (rs79453636) as associated with CRC in AA, independent of rs3802842, that has not been replicated in other populations [60]. Neither rs79453636, nor any SNPs in LD with it, was identified as a colon *cis*-eQTL in the present study.

Enrichment of colon *cis*-eQTL among trait-associated variants as shown in Table 4 is consistent with other studies [44,61-63] and adds further evidence for the usefulness of eQTL in improving power to detect significant associations from GWAS. Enrichment of colon *cis*-eQTL was observed in diseases with colonic involvement (IBD and CRC), as well as in T2D and BMI. These results raise the intriguing hypothesis that gene expression in the colon could be functionally linked to diabetes and BMI, though further work is needed to identify specific mechanisms. No significant enrichment was found for colon *cis-*eQTL among variants associated with autoimmune disorders, cancer or psychiatric disorders. Taken together, these results support the conclusion that eQTL most relevant to disease show clear tissue specificity, highlighting the importance of creating comprehensive eQTL catalogs in diverse tissue types [64].

A substantial proportion of the gene targets of the significant colon *cis*-eQTL were also found to be *cis*-regulated target genes in other tissue types notably liver and ileum – two tissues closely related to the colon (Table 3). The percentage overlap of *cis*-eQTL target genes between colon and other tissue types was higher at more stringent FDR thresholds for *cis*-eQTL associations, which was expected because increased stringency should filter out false positive results. The number of significant target genes shared between studies will also be affected by sample size, significance thresholds and genotyping platforms. Therefore, the actual percentage values of overlap should be regarded as rough estimates of the actual extent of overlap. The high cross-tissue replicability of the colon *cis*-eQTL is consistent with the observation here of active histone mark enrichment in non-colon tissue types as well as findings from other studies that showed that a large number of *cis*-eQTL are shared across tissues [42,46,65]. The extent to which eQTL have cell-type or context-specificity in the human colon remains to be investigated but could yield important information for further functional characterization.

Consistent with the hypothesis that eQTL have regulatory functions, enrichment was noted for colon *cis-*eQTL near TSS similar to findings in other tissues [34,39,46-48]. Moreover, *cis*-eQTL were significantly enriched for histone marks associated with active chromatin (H3K4me1, H3K4me3, H3K9ac and H3K36me3) but not with transcriptionally inactive chromatin (H3K9me3 and H3K27me3) in various tissues and in a CRC cell line. These findings are consistent with the widely accepted theory that *cis*-eQTL act by affecting transcript stability or the rate of transcript degradation [39,66-68], and other studies that have also noted enrichment of eQTL among activating *cis*-elements but not in regions of repressed chromatin [69-71].

A substantial proportion of colon *cis*-eQTL in this study were highly differentiated between European and African populations (F_ST_ > 0.25) as shown in Figure 3. The degree of population differentiation among these eQTL suggests that they may well reflect local adaptation to environment. A similar finding was previously described for LCL eQTL [72]. It remains to be investigated whether such colon eQTL, which exhibit large allele frequency differences between European and African populations, contribute to inter-ethnic gene expression differences in the colon that translate into differences in disease risk. Integration of GWAS results with population genetic approaches may prove instrumental in understanding the genetic basis of differences in IBD and CRC risk between Europeans and AA [73-76].

A limitation of this study is the moderate sample size, which reduces power to detect significant eQTL associations (especially those acting in *trans*), thereby resulting in a high false negative rate. Further investigation in a larger cohort is likely to result in more significant eQTL associations, and is warranted to confirm findings from this study. Another caveat of this study is that the GWAS SNPs used in the analyses were identified in Europeans, whereas the eQTL were identified in AA. Although many of the variants identified through GWAS in European populations generalize to other populations, there are differences in genetic susceptibility between populations, due to several factors including epistasis, gene-environment interactions, population-specific polymorphisms and differential LD patterns [77]. Therefore, it is possible that there are differences in genetic associations between European and AA populations. As IBD and CRC GWAS in populations of African descent become available, the overlap between eQTL identified in this study and GWAS SNPs in these populations should be investigated.

## Conclusions

In the present study, a comprehensive map of eQTL in the healthy colon of AA was generated as a resource for genetic studies of colonic diseases. The analysis showing the enrichment of colon eQTL among SNPs associated with colonic diseases supported the usefulness of colon eQTL as a tissue-specific tool to improve understanding of colonic disease susceptibility. The utility of colon eQTL for studying the genetic basis of inter-ethnic differences in colonic disease risk was demonstrated by showing their enrichment for SNPs that exhibit high allele frequency differences between European and African populations. These SNPs could mediate population-specific gene expression responses, which could translate into differences in disease risk.

This study offers novel insights into the functional basis of genetic susceptibility for colonic inflammatory and malignant diseases and provides a tissue-specific resource for future studies. Characterization of the genetic architecture of gene regulation in the human colon informs the functional impact of GWAS variants and could benefit understanding of the biological differences in colonic disease between different ethnic groups. The findings in this study demonstrate that eQTL are important in the susceptibility to inflammatory and malignant diseases of the colon, underscoring the utility of eQTL mapping for elucidating the genetics and biology underlying colonic diseases. Furthermore the eQTL map presented here could benefit understanding of the biological differences in colonic disease between different ethnic groups. This is especially important given that there is a paucity of genetic studies of colonic diseases in AA populations and our understanding of the genetics and etiology of these diseases is based on Europeans, which may not be applicable to other populations. Further efforts should be made to intersect our eQTL data with IBD and CRC genetic studies, for a better understanding of the genetic mechanisms and inter-ethnic differences in these diseases.

## Methods

### Subjects

A total of 48 AA subjects were included. All subjects underwent colonoscopy at the University of Illinois Chicago for screening purposes. Blood samples were obtained at the time of colonoscopy. Colonic biopsies were obtained using standard forceps (Boston Scientific, Natick, MA) in all subjects at 20 cm from the anal verge at the recto-sigmoid junction. Biopsies were placed immediately into RNAlater (Life Technologies Corporation, Grand Island, NY), and cryopreserved according to the manufacturer’s instructions. Subjects’ mean age (standard deviation) was 59.0 years (8.5). While the intention was to include only males in order to reduce heterogeneity, two female subjects were included due to sample mislabeling. Written informed consent was obtained from participants.

### Ethics statement

This research was approved by the Institutional Review Boards at the University of Illinois at Chicago and the University of Chicago.

### Genotyping and imputation

Colon samples from study subjects were genotyped on the Affymetrix Axiom Genome-Wide Pan-African array that includes 2,217,402 probes (Affymetrix, Santa Clara, CA). Genotypes were called using the Illumina Genome Studio software package (Illumina, San Diego, CA). QC filters applied to the data are outlined in Figure 1. The analysis was limited to SNPs that map to unique chromosomal locations based on the latest annotation file for the genotyping array (Release 33). Markers with <95% genotype call rates, MAF < 0.05 or HWE p < 10^−6^ were excluded from analysis. Markers with HWE p < 10^−4^ for which there were no heterozygotes or homozygotes for the minor allele, as well as markers with HWE p < 10^−3^ for which there were <6 heterozygotes or homozygotes for the minor allele were also excluded. Finally, all non-autosomal SNPs were omitted. The final genotyped dataset contained 1,492,955 autosomal SNPs in 48 subjects with an average call rate of 99.69%.

Imputation was performed with IMPUTE2 version 2.2.2 [78,79] using the 1000 Genomes Phase I integrated variant set release (v3) (https://mathgen.stats.ox.ac.uk/impute/impute_v2.html) [51] as reference panel. IMPUTE2 uses a reference panel of known haplotypes and a fine-scale recombination map to infer missing genotypes in a study dataset that has been typed at a relatively sparse set of markers. IMPUTE2 assigns each imputed SNP an INFO score on the basis of imputation quality. INFO scores can take values from 0 to 1, where values closer to 1 indicate that there is little uncertainty in the imputed genotype. Imputed SNPs with MAF < 0.05 and IMPUTE2-info score < 0.5 were excluded from the analysis, leaving 8,400,922 imputed SNPs in 48 subjects. Imputed genotypes were coded as allelic dosages (i.e. estimated fractional counts of the non-reference allele ranging continuously from 0 to 2).

### Transcriptional profiling

RNA was extracted from manually ground tissue using the Maxwell 16 Tissue LEV Total RNA Purification Kit (Promega, Fitchburg, WI) for automated purification on the Maxwell 16 Instrument (Promega, Fitchburg, WI). Gene expression levels were measured using the Illumina HT-12v4 Expression BeadChip whole-genome expression array containing 47,231 gene probes (Illumina, San Diego, CA), according to manufacturer’s instructions. The Illumina GenomeStudio software was used to extract the signal intensity for each probe, which were then further preprocessed using the lumi package of Bioconductor (http://www.bioconductor.com) [80,81] in R version 3.0.2. The preprocessing included background correction of the expression data, followed by variance stabilization transformation (VST), log2-transformation and quantile-normalization.

BLAST-like alignment tool (BLAT) pairwise sequence alignment algorithm was used to map the probe sequences onto the RefSeq transcriptome (downloaded from the UCSC Genome Browser: http://hgdownload.cse.ucsc.edu/goldenPath/hg19/bigZips). All probes mapping to multiple Entrez Gene IDs were omitted and only probes that map uniquely to autosomal genes with no mismatches were included. Probes that encompass a common SNP (MAF ≥ 0.05) in either 1000 Genomes AFR or EUR populations were further excluded, leaving 25,610 probes with unique perfect matches to autosomal genes that do not contain common SNPs. Furthermore only probes with an Illumina detection p < 0.01 in at least one individual were included in further analysis.

Eight individuals were identified as outliers based their poor clustering with other subjects on gene expression heat map (Additional file 2). There was no correlation between any of the demographic and clinical characteristics and outlier status. The separation of the outliers from the rest of the samples seemed to be driven by systematic differences in gene expression rather than by expression differences of only a few genes. Four of the outlier samples had low RNA integrity numbers (RIN) (<8). As the outliers with RIN > 8 seemed to cluster with the four outliers with the low RIN scores, the outliers were likely driven by poor RNA quality. Upon the exclusion of the eight outlier samples, there were no further outliers on the gene expression heat map. The outliers were excluded from the gene expression analysis prior to the microarray pre-processing steps in Bioconductor. After the removing probes that were not significantly expressed, the final expression dataset included 16,252 probes interrogating 12,363 unique genes in 40 subjects. The gene expression data is available at Gene Expression Omnibus (http://www.ncbi.nlm.nih.gov/projects/geo) with the accession number GSE 56789.

The linear model that is used in the eQTL analysis assumes that the expression values for each probe follow a normal distribution, and it may be sensitive to the presence of outliers and non-normality. Thus the log2-transformed and quantile-normalized expression values for each probe were transformed into a standard normal distribution using the rank-based inverse normal transformation implemented in the qqnorm function in R prior to the eQTL analysis.

Colon biopsies were composed of primarily epithelial cells with a small amount of stromal cells. At least 2 biopsies from the colon were used per individual to minimize tissue heterogeneity. Overall, expression of an epithelial marker *E-cadherin* (*CDH1*) did not show significant inter-individual variation when normalized to a housekeeping gene *GAPDH* across the 40 individuals (data not shown).

### Ancestry estimates

PCA of the genotype data was used to quantify the proportions of European and African ancestry in the AA subjects. Samples from HapMap ASW, CEU, and YRI populations were used as reference populations. PCA analysis was performed in EIGENSTRAT [82] using an LD-pruned dataset of 48,553 SNPs. The first PC from the analysis was included as a covariate to account for ancestry in subsequent analyses.

### eQTL mapping

eQTL mapping was performed using the Matrix eQTL R package [83]. Associations between SNP genotype and probe expression level were analyzed using a linear regression model with additive genotype effects. *Cis*-eQTL were defined as associations between SNPs within 1 mega base pair (Mb) of the TSS or transcription end site (TES) of a gene. *Trans*-eQTL were defined as association signals from SNPs located greater than 1 Mb from the TSS and TES of a gene or on a different chromosome. FDR calculations were performed separately for *cis-* and *trans*-eQTL in Matrix eQTL according to the Benjamini and Hochberg method [84]. A PCA-based approach was used to correct for confounding variability due to hidden factors in the gene expression data [85] as outlined in the Supplementary Methods (Additional file 8).

To determine the number of independent *cis*-eQTL associations at the different FDR thresholds, a stepwise association model was applied that involves repeating the eQTL association analysis conditioned on the most significant *cis*-eQTL for each gene expression probe, until there were no more significant *cis*-associations left as described in the Supplementary Methods (Additional file 8).

### Colon *cis*-eQTL associated with colonic diseases

All NHGRI GWAS SNPs associated with colonic diseases (CD, UC and CRC) that were either significant *cis*-eQTL (FDR < 0.10) or in high LD with a *cis*-eQTL were identified. All SNPs in high LD (r^2^ ≥ 0.8) with the *cis*-eQTL were obtained from SNAP database (http://www.broadinstitute.org/mpg/snap) [86] using CEU as the reference. Associations were visualized using box plots that show expression as a function of eQTL genotype. For details, refer to Supplementary Methods (Additional file 8).

### Evaluation of *cis*-eQTL enrichment among trait-associated SNPs

The enrichment of significant *cis*-eQTL among SNPs associated with various complex traits and diseases were investigated. For the enrichment analyses, only trait-associated SNPs that were among the 8,400,922 SNPs analyzed were considered. Trait-associated GWAS SNPs were downloaded from the NHGRI Catalog of Published GWAS using the default p-value threshold of 10^−5^ [87]. SNPs that were not identified in populations of European descent were excluded. The complex traits/diseases were classified into 5 broad categories: (1) autoimmune disorders (celiac disease [n = 57], psoriasis [n = 35] and RA [n =54]) (2) cancers (breast cancer [n = 100], prostate cancer [n = 121] and melanoma [n = 16]), (3) colonic diseases (CD [n = 251], UC [n = 185] and CRC [n = 40]), (4) BMI (n = 100), lipid traits (n = 210) and T2D (n = 65) and (5) psychiatric disorders (ADHD [n = 179], BD [n = 257] and schizophrenia [n = 247]). The number of GWAS SNPs for each trait that fit the aforementioned criteria and were thus included in the enrichment analyses are indicated by n and listed inside the brackets.

Simulation-based enrichment analyses were conducted as previously described [44], to evaluate if the trait-associated SNPs were enriched for colon *cis*-eQTL. Briefly, 1,000 randomized SNP sets were generated sampled without replacement from the 8,400,922 study SNPs. Since there was an overrepresentation of higher-frequency variants among the significant *cis*-eQTL compared to all study SNPs (Additional file 10), SNPs were matched on MAF distribution. SNPs were also matched on distance to the nearest TSS since *cis*-eQTL were more enriched around TSS compared to other SNPs (Additional file 11). For each sampled SNP set and the set of NHGRI SNPs, the number of SNPs that were also *cis*-eQTL was determined. The relatively relaxed significance threshold of p < 0.001 was used for eQTL p-values since the numbers of trait-associated *cis-*eQTL were too low to quantify enrichment at higher thresholds of significance. An empirical p-value for *cis*-eQTL enrichment was obtained based on the simulations, calculated as the proportion of sampled SNP sets in which the *cis*-eQTL count exceeds the actual *cis*-eQTL count observed in the NHGRI SNPs.

### Bioinformatic analysis of *cis*-eQTL

The genomic locations of *cis-*eQTL were characterized by plotting their position relative to the TSS of genes. The enrichment of histone marks among significant *cis*-eQTL at an FDR threshold of 0.10 was evaluated using a modified version of the simulation-based strategy that was used to test for eQTL enrichment among trait-associated SNPs. Briefly, the number of SNPs in histone marks peaks among 1,000 sets of independent *cis*-eQTL were compared to that among 1,000 randomized SNP sets, matched based on the distributions of MAF and distance from the nearest TSS to the independent *cis*-eQTL. For details and adaptations, see Supplementary Methods (Additional file 8).

### Comparative analysis with *cis*-eQTL from other tissues

The overlap between *cis-*eQTL in the colon and *cis*-eQTL in other tissue was determined using the Genotype-Tissue Expression (GTEx) eQTL browser (available at http://www.ncbi.nlm.nih.gov/gtex/GTEX2/gtex.cgi), which allows the query of eQTL results from multiple previous studies by SNP or gene name. To evaluate the overlap of the colon *cis*-eQTL with *cis*-eQTL from another tissue, the GTEx eQTL browser was queried to obtain *cis*-eQTL p-values for genes regulated by a significant colon *cis*-eQTL identified in this study (at four different FDR thresholds: 0.01, 0.05, 0.10 and 0.20). The number of genes that were also *cis*-regulated, defined as within 1 Mb of a gene, in other studies was determined at a p-value cut-off of 10^−5^. These studies included the following tissue types: LCLs [37], liver [35] and brain [48]. *Cis*-eQTL target genes were considered to be overlapping between the colon and another tissue if both studies had at least one *cis*-eQTL for that target gene. Overlap of colon with skin *cis*-eQTL (defined as within 1 Mb of a gene and with p < 10^−5^) [46] and colon with ileum *cis*-eQTL (defined as within 50 kilo base pairs (kb) of a gene and with p *<* 10^−5^) [49] was also investigated using the same approach. Skin *cis*-eQTL data was obtained from the University of Michigan, Center for Statistical Genetics website (http://www.sph.umich.edu/csg/junding/eQTL/). Ileum *cis*-eQTL data was downloaded from the Additional file of the article [49]. For each tissue type, the total number of target genes that overlap between colon and other tissues was reported along with the number of overlapping target genes that are associated with the same *cis*-eQTL SNP (or a proxy with r^2^ ≥ 0.8) in both tissues. The number of SNP-gene pairs that overlap between the present study and other eQTL studies was reported as well. Our rationale for focusing on the overlap of eQTL target genes rather than the overlap of eQTL SNPs, is that genes are more comparable and less biased across different studies that use different genotyping platforms with different marker sets.

### Fixation index analysis of *cis*-eQTL

To estimate the level of genetic differentiation between European and African populations at the colon *cis*-eQTL, F_ST_ values for all colon *cis*-eQTL (n = 14,177; FDR < 0.20) were calculated by using Weir and Cockerham’s unbiased estimator [88]. For the F_ST_ calculations, genotype data for EUR and AFR populations from the 1000 Genomes Project was used [54]. To test whether the number of independent colon *cis*-eQTL that show high population differentiation between European and African populations is greater than that expected by chance, we performed a simulation-based analysis which we describe in detail in the Supplementary Methods (Additional file 8). SNPs that show high population differentiation between EUR and AFR were defined as those with F_ST_ > 0.25. The number of highly population differentiated SNPs among all 1,000 randomized SNP sets was compared to the number of highly population differentiated SNPs among the sets of independent *cis*-eQTL using the Mann–Whitney test. High F_ST_ SNPs (F_ST_ > 0.10) were identified among *cis*-eQTL that were associated with CD, UC or CRC and allele frequency plots showing the worldwide distributions of ancestral and derived alleles in human populations were constructed. The data used in the generation of these plots are described in detail in a previous publication [89].

## Additional files

Additional file 1: Figure S1. PCA plot of the genotype data from the study samples along with HapMap reference populations. The 48 African American subjects cluster with HapMap African American samples on the principal components analysis (PCA) plot, indicating mixed African and European ancestry. A PCA-based analysis was performed in EIGENSTRAT to estimate the proportions of European and African ancestry for each subject (represented by PC1). HapMap populations of African ancestry in Southwest USA (ASW), Utah residents of Northern and Western European ancestry (CEU) and Yoruba in Ibadan, Nigeria (YRI) were used as references in the PCA plot and are color-coded as indicated in the legend. PC1 was included as a covariate in the eQTL analysis to account for ancestry. Additional file 2: Figure S2. Heat map visualization and hierarchical clustering of the gene expression data. Study samples are organized based on the similarity of log2-transformed and quantile-normalized gene expression levels across all probes evaluated in the study. The heat maps represent the pairwise correlation matrix of similarity among the study subjects across all probes. Each row and column represents one of the subjects. The color scale indicates the degree of correlation from lowest (white) to highest (red). (A) Heat map corresponding to the gene expression data for the 48 subjects prior to the removal of outliers. The first eight subjects from the left side of the plot were deemed as outliers and excluded from the eQTL analysis. (B) Heat map corresponding to the gene expression data for the 40 remaining subjects after the removal of outliers. Additional file 3: Figure S3. The numbers of significant *cis*-eQTL probes were maximized with the inclusion of the first five PCs of the gene expression data as covariates. Each of the first 20 PCs from the gene expression data were sequentially included as covariates in the analysis and the numbers of gene expression probes that were associated with a *cis*-eQTL were determined for each at two different FDR thresholds for SNP-gene associations. The maximum numbers of gene expression probes associated with a *cis-*eQTL were obtained when the first five PCs from the gene expression data were used as covariates in the eQTL analysis. Thus the first five PCs were included as covariates in subsequent analyses, to correct for unmeasured variation in the gene expression data. Additional file 4: Figure S4. The colon eQTL dataset is enriched for *cis*- but not *trans*-eQTL. (A) The Q-Q plot shows the eQTL association p-values for all SNP-probe pairs (8,400,922 SNPs x 16,252 probes) evaluated in the study. Red dots represent *cis-* and blue dots represent *trans-*association p-values. The gray line is the identity line (y = x) representing the null distribution under which there is no association between SNP genotype and probe expression level. *Cis*-association p-values deviate strongly from the identity line, demonstrating an enrichment of significant associations. There is no such enrichment for *trans*-associations. (B) The histogram shows the eQTL association p-values for all *cis* SNP-probe pairs. There is a clear enrichment of *cis-*associations with small p-values. Additional file 5: Figure S5.
*Cis*-eQTL cluster roughly symmetrically around TSS. The scatter plot depicts the distribution of *cis*-eQTL relative to TSS. Each dot represents the most significant *cis*-associated SNP for each gene expression probe. –log10(p-value) for the SNP-probe association (y-axis) is plotted against the base pair (bp) distance of the associated SNP from the TSS of the transcript that the probe is interrogating (x-axis). Negative and positive values of the distance denote SNPs 5′ and 3′ of TSS (set at 0), respectively. The majority of significant *cis*-eQTL are found within 100 kb of TSS. Additional file 6: Figure S6. Human populations across the world differ in the designations of ancestral and derived alleles as major alleles for UC-associated colon *cis*-eQTL rs9847710. rs9847710 exhibits a relatively high level of population differentiation between 1000 Genomes EUR and AFR populations (F_ST_ > 0.10). The plot shows the distribution of rs9847710 alleles in multiple populations worldwide. The haplotype frequencies for different human populations are depicted in pie charts where the ancestral allele (C) is shown in blue and the derived allele (T) is shown in orange. Additional file 7: Figure S7. SNPs associated with diseases that do not involve the colon are not enriched for colon *cis*-eQTL. This is similar to Figure 4, except the plots depict the enrichment of colon *cis*-eQTL among SNPs associated with (A) autoimmune disorders, (B) cancers and (C) psychiatric disorders. There is no significant enrichment for colon *cis*-eQTL among SNPs associated with these disorders. Additional file 8:
**Supplementary Methods.**
Additional file 9: Figure S8.
*Cis*-eQTL are enriched for active but not repressive histone marks in a colorectal adenocarcinoma cell line. This is similar to Figure 5, except the histone mark data is from a colorectal adenocarcinoma cell line (Caco-2) and only two active histone marks (H3K4me3 and H3K36me3) and one repressive histone mark (H3K27me3) are depicted. Colon *cis*-eQTL are enriched for active histone marks with the enrichment reaching statistical significance for H3K4me3 (p < 1.0 × 10^-6^) and being highly suggestive for H3K36me3 (p = 0.060). There is no statistically significant enrichment of the repressive histone mark H3K27me3. Additional file 10: Figure S9. MAF distribution of colon *cis*-eQTL is skewed towards higher frequencies compared with the complete set of study SNPs. MAF distribution of the complete set of study SNPs (top graph) is presented, along with the MAF distributions of *cis*-eQTL at four different FDR thresholds. The skew towards higher frequencies becomes more pronounced as the FDR threshold becomes more stringent. Additional file 11: Figure S10. Colon *cis*-eQTL are closer to TSS than other SNPs. The histograms show the distributions of the base pair (bp) distances to the nearest TSS for all study SNPs that are within 1 mega base pair (Mb) of a TSS and significant *cis*-eQTL at various FDR thresholds. Negative distances refer to SNPs upstream (5′) of the TSS (set at 0) while positive distances refer to those downstream (3′).

## Abbreviations

AA: African American
*ADCY3*: Adenylate cyclase 3
ADHD: Attention deficit hyperactivity disorder
AFR: 1000 Genomes Project African population
ASW: African ancestry in Southwest USA
BD: Bipolar disorder
BLAT: BLAST-like alignment tool
BMI: Body mass index
CD: Crohn’s disease
CEU: Utah residents with Northern and Western European ancestry
ChIP: Chromatin immunoprecipitation
*COLCA2*: Colorectal cancer associated 2
CRC: Colorectal cancer
eQTL: Expression quantitative trait loci
*ERAP2*: Endoplasmic reticulum aminopeptidase 2
EUR: 1000 Genomes Project European population
FDR: False discovery rate
F_ST_: Fixation index
GEO: Gene Expression Omnibus
GTEx: Genotype-Tissue Expression
GWAS: Genome-wide association study
HWE: Hardy-Weinberg equilibrium
IBD: Inflammatory bowel disease
*INPP5E*: Inositol polyphosphate-5-phospatase
Kb: Kilo base pair
LCL: Lymphoblastoid cell line
LD: Linkage disequilibrium
MAF: Minor allele frequency
Mb: Mega base pair
MHC1: Major histocompatibility complex class I
mRNA: Messenger ribonucleic acid
NHGRI: National Human Genome Research Institute
*NXPE1*: Neuroexophilin and PC-esterase domain family member 1
PC: Principal component
PCA: Principal components analysis
QC: Quality control
Q-Q: Quantile-quantile
RA: Rheumatoid arthritis
RIN: RNA integrity number
*REXO2*: RNA exonuclease 2
*SFMBT1*: Scm-like with four MBT domains 1
SNP: Single nucleotide polymorphism
T2D: Type 2 diabetes
TES: Transcription end site
TSS: Transcription start site
*UBA7*: Ubiquitin-like modifier activating enzyme 7
UC: Ulcerative colitis
VST: Variance stabilizing transformation
YRI: Yoruba in Ibadan, Nigeria
