# Supplementary figures and images for: Enrichment of inflammatory bowel disease and colorectal cancer risk variants in colon expression quantitative trait loci
Source: BMC Genomics. 2015 Feb 27;16(1):138. doi: 10.1186/s12864-015-1292-z (PMC4351699; doi:10.1186/s12864-015-1292-z)

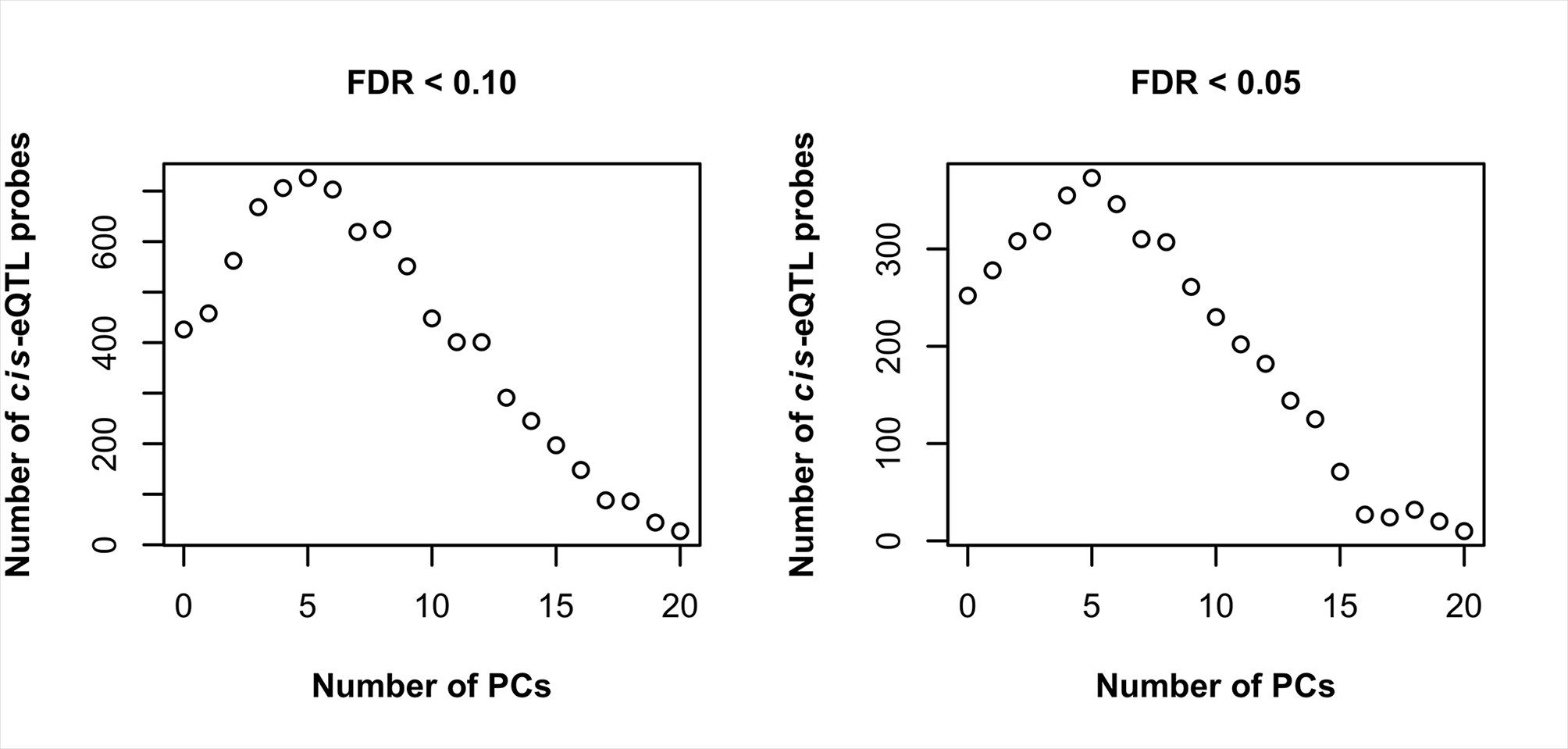

Supplement: Additional file 3: Figure S3. — The numbers of significant cis-eQTL probes were maximized with the inclusion of the first five PCs of the gene expression data as covariates. Each of the first 20 PCs from the gene expression data were sequentially included as covariates in the analysis and the numbers of gene expression probes that were associated with a cis-eQTL were determined for each at two different FDR thresholds for SNP-gene associations. The maximum numbers of gene expression probes associated with a cis-eQTL were obtained when the first five PCs from the gene expression data were used as covariates in the eQTL analysis. Thus the first five PCs were included as covariates in subsequent analyses, to correct for unmeasured variation in the gene expression data. [file 12864_2015_1292_MOESM3_ESM.tiff]

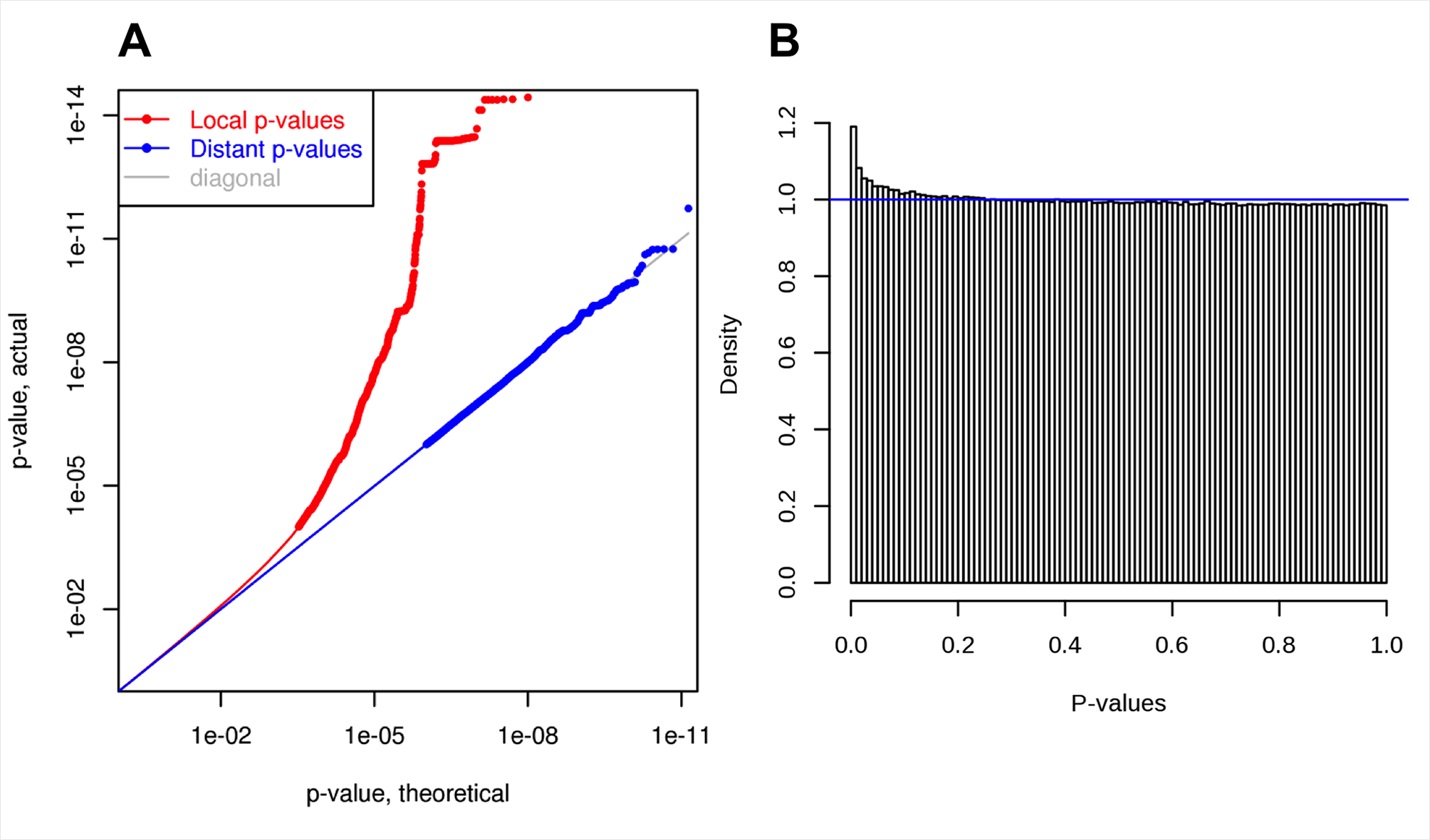

Supplement: Additional file 4: Figure S4. — The colon eQTL dataset is enriched for cis- but not trans-eQTL. (A) The Q-Q plot shows the eQTL association p-values for all SNP-probe pairs (8,400,922 SNPs x 16,252 probes) evaluated in the study. Red dots represent cis- and blue dots represent trans-association p-values. The gray line is the identity line (y = x) representing the null distribution under which there is no association between SNP genotype and probe expression level. Cis-association p-values deviate strongly from the identity line, demonstrating an enrichment of significant associations. There is no such enrichment for trans-associations. (B) The histogram shows the eQTL association p-values for all cis SNP-probe pairs. There is a clear enrichment of cis-associations with small p-values. [file 12864_2015_1292_MOESM4_ESM.tiff]

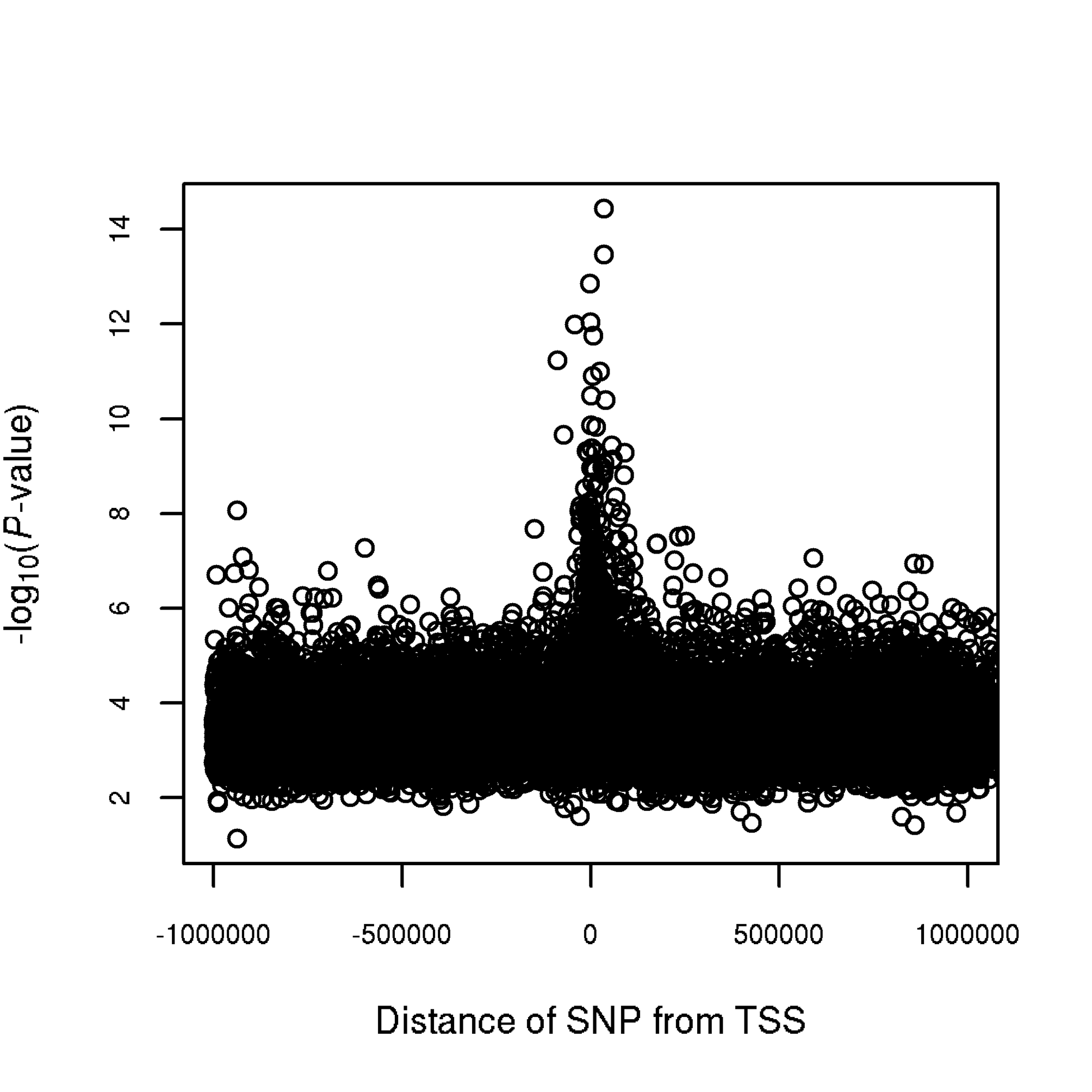

Supplement: Additional file 5: Figure S5. — Cis-eQTL cluster roughly symmetrically around TSS. The scatter plot depicts the distribution of cis-eQTL relative to TSS. Each dot represents the most significant cis-associated SNP for each gene expression probe. –log10(p-value) for the SNP-probe association (y-axis) is plotted against the base pair (bp) distance of the associated SNP from the TSS of the transcript that the probe is interrogating (x-axis). Negative and positive values of the distance denote SNPs 5′ and 3′ of TSS (set at 0), respectively. The majority of significant cis-eQTL are found within 100 kb of TSS. [file 12864_2015_1292_MOESM5_ESM.tiff]

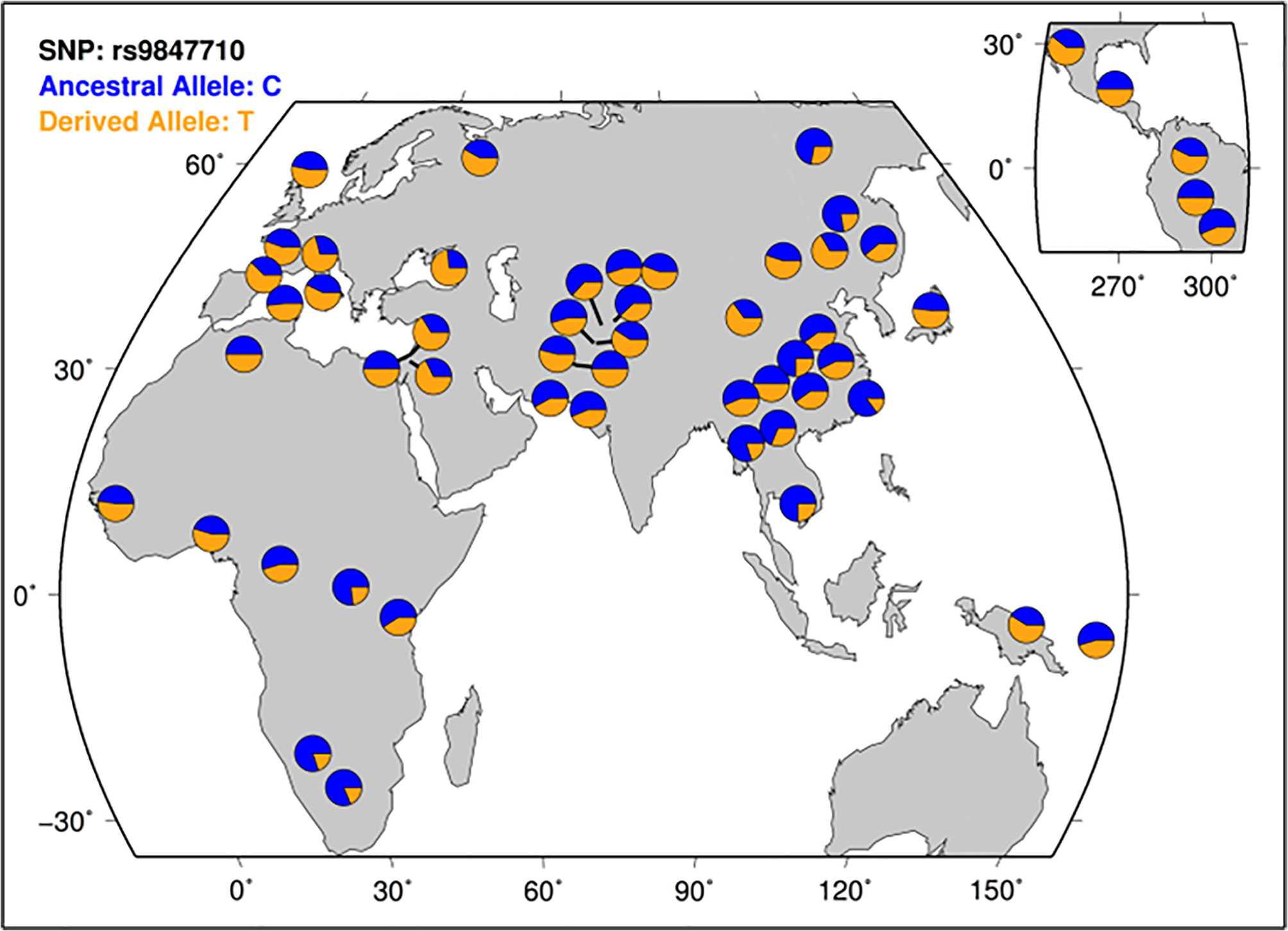

Supplement: Additional file 6: Figure S6. — Human populations across the world differ in the designations of ancestral and derived alleles as major alleles for UC-associated colon cis-eQTL rs9847710. rs9847710 exhibits a relatively high level of population differentiation between 1000 Genomes EUR and AFR populations (FST > 0.10). The plot shows the distribution of rs9847710 alleles in multiple populations worldwide. The haplotype frequencies for different human populations are depicted in pie charts where the ancestral allele (C) is shown in blue and the derived allele (T) is shown in orange. [file 12864_2015_1292_MOESM6_ESM.tiff]

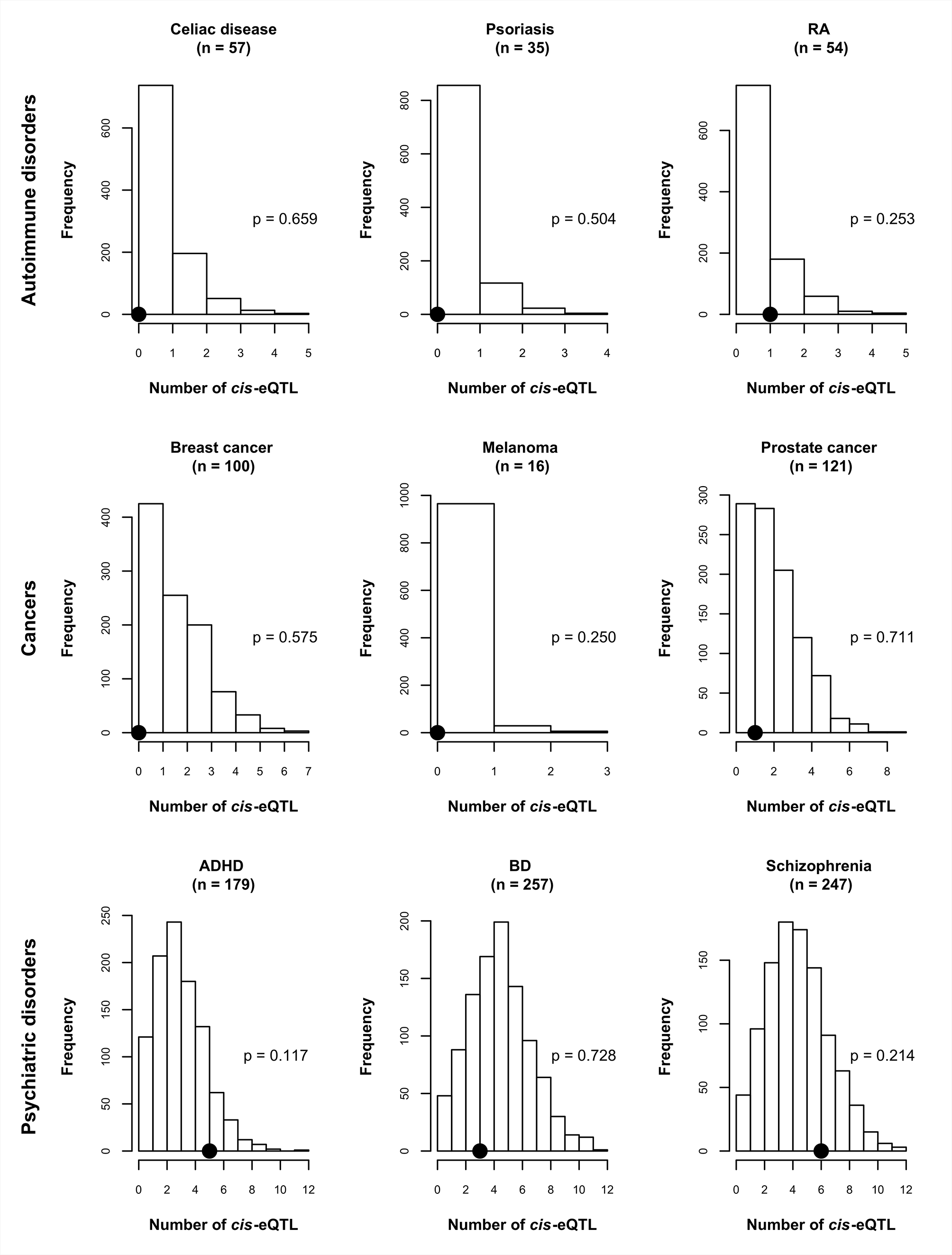

Supplement: Additional file 7: Figure S7. — SNPs associated with diseases that do not involve the colon are not enriched for colon cis-eQTL. This is similar to Figure 4, except the plots depict the enrichment of colon cis-eQTL among SNPs associated with (A) autoimmune disorders, (B) cancers and (C) psychiatric disorders. There is no significant enrichment for colon cis-eQTL among SNPs associated with these disorders. [file 12864_2015_1292_MOESM7_ESM.tif]

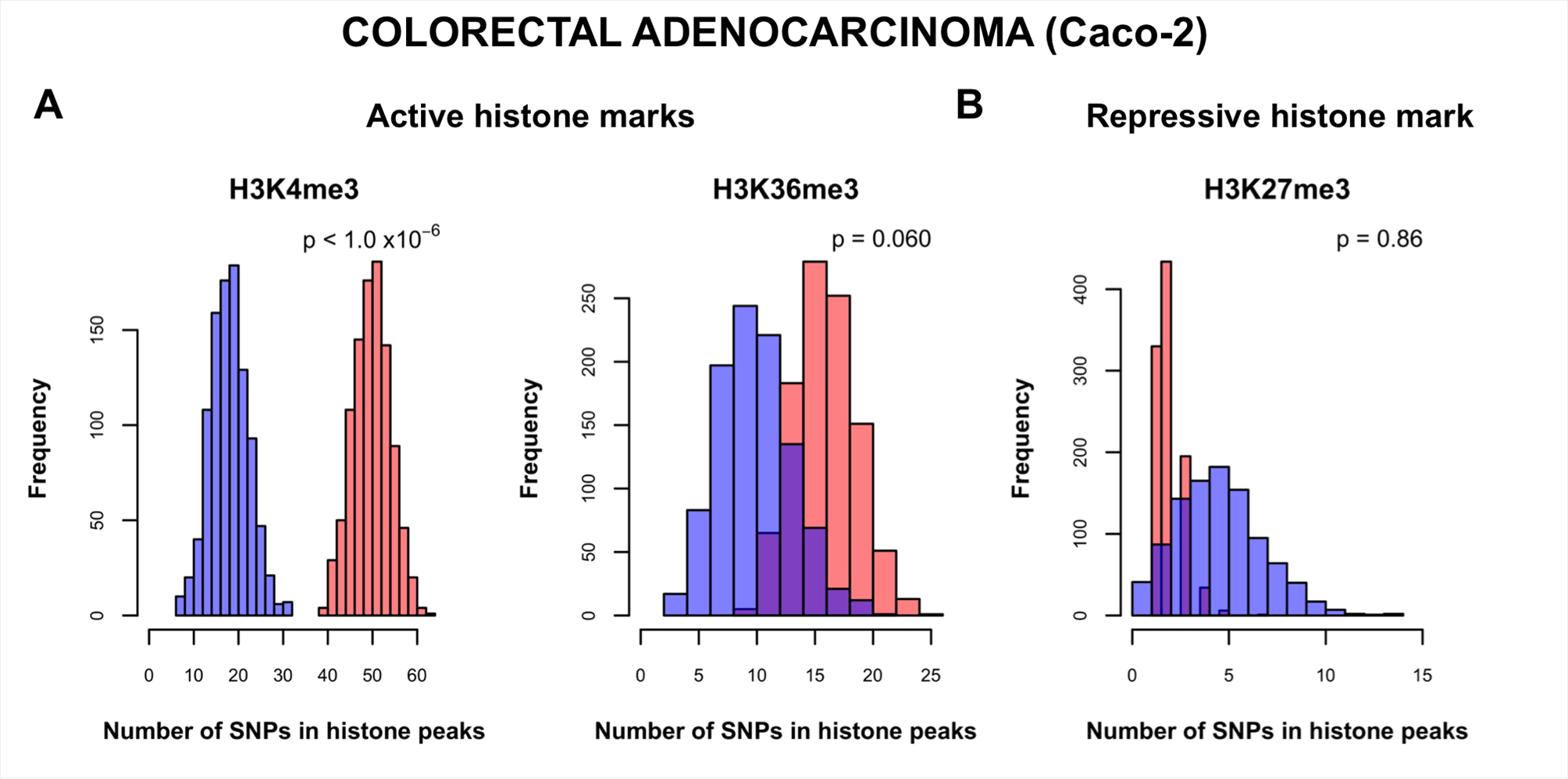

Supplement: Additional file 9: Figure S8. — Cis-eQTL are enriched for active but not repressive histone marks in a colorectal adenocarcinoma cell line. This is similar to Figure 5, except the histone mark data is from a colorectal adenocarcinoma cell line (Caco-2) and only two active histone marks (H3K4me3 and H3K36me3) and one repressive histone mark (H3K27me3) are depicted. Colon cis-eQTL are enriched for active histone marks with the enrichment reaching statistical significance for H3K4me3 (p < 1.0 × 10^-6^) and being highly suggestive for H3K36me3 (p = 0.060). There is no statistically significant enrichment of the repressive histone mark H3K27me3. [file 12864_2015_1292_MOESM9_ESM.tiff]

All Study SNPs (n = 8,400,922)

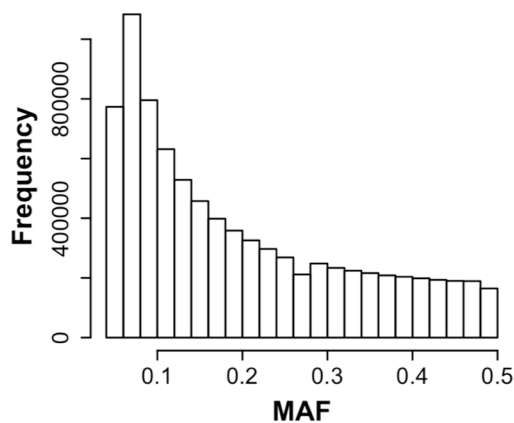

*cis*-eQTL (FDR < 0.20, n = 14,177)

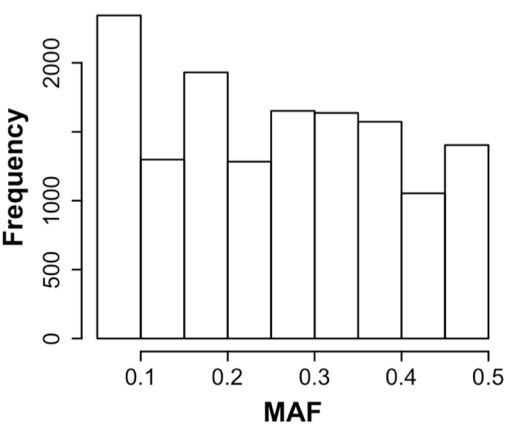

*cis*-eQTL (FDR < 0.10, n = 8,222)

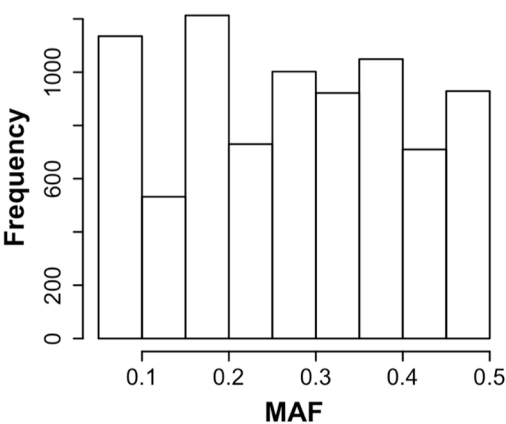

*cis*-eQTL (FDR < 0.05, n = 5,181)

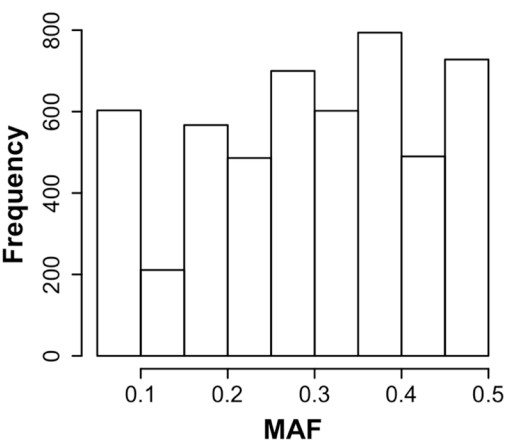

*cis*-eQTL (FDR < 0.01, n = 1,941)

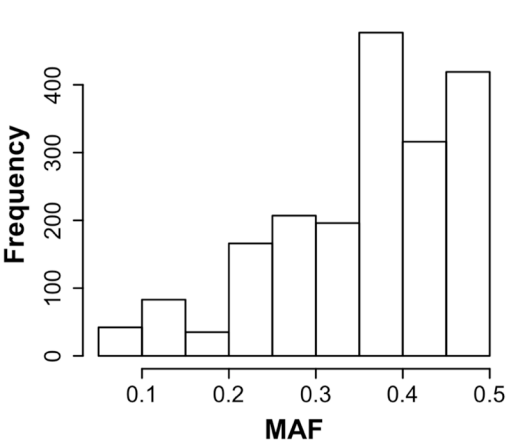

Supplement: Additional file 10: Figure S9. — MAF distribution of colon cis-eQTL is skewed towards higher frequencies compared with the complete set of study SNPs. MAF distribution of the complete set of study SNPs (top graph) is presented, along with the MAF distributions of cis-eQTL at four different FDR thresholds. The skew towards higher frequencies becomes more pronounced as the FDR threshold becomes more stringent. [file 12864_2015_1292_MOESM10_ESM.pdf]

**All SNPs within 1 Mb of a TSS  
(n = 7,689,243)**

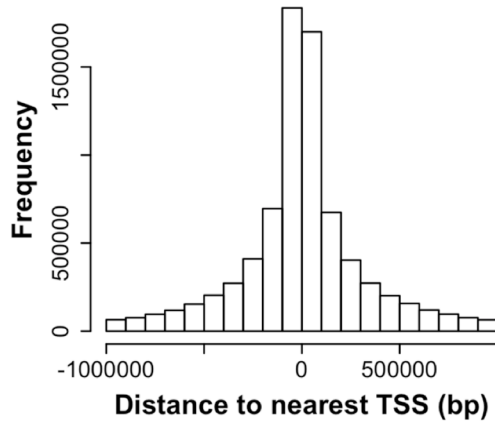

***cis*-eQTL (FDR < 0.20, n = 14,177)**

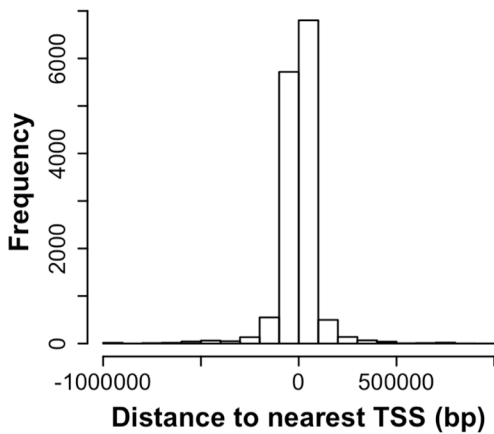

***cis*-eQTL (FDR < 0.10, n = 8,222)**

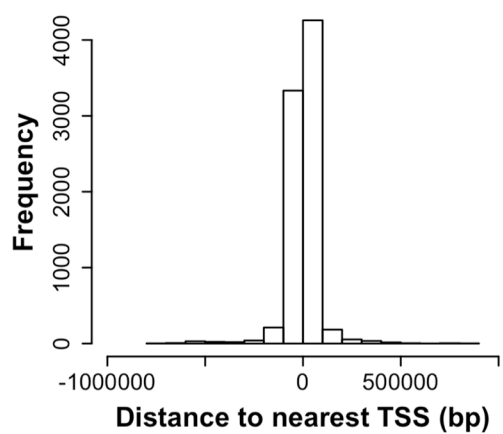

***cis*-eQTL (FDR < 0.05, n = 5,181)**

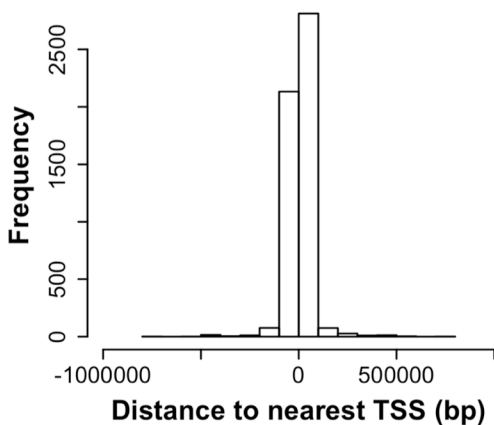

***cis*-eQTL (FDR < 0.01, n = 1,941)**

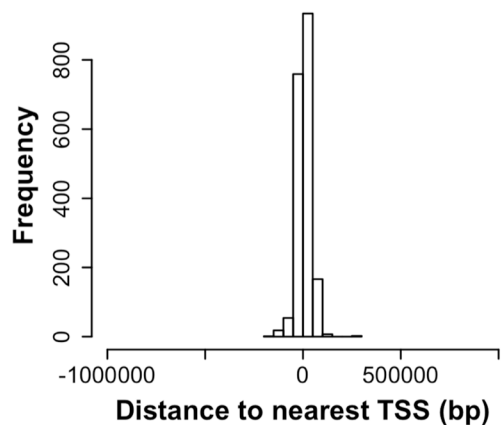

Supplement: Additional file 11: Figure S10. — Colon cis-eQTL are closer to TSS than other SNPs. The histograms show the distributions of the base pair (bp) distances to the nearest TSS for all study SNPs that are within 1 mega base pair (Mb) of a TSS and significant cis-eQTL at various FDR thresholds. Negative distances refer to SNPs upstream (5′) of the TSS (set at 0) while positive distances refer to those downstream (3′). [file 12864_2015_1292_MOESM11_ESM.pdf]
