# Supplementary material for: Enrichment of inflammatory bowel disease and colorectal cancer risk variants in colon expression quantitative trait loci
Source: BMC Genomics. 2015 Feb 27;16(1):138. doi: 10.1186/s12864-015-1292-z (PMC4351699; doi:10.1186/s12864-015-1292-z)
Supplement: Additional file 8: — Supplementary Methods. [file 12864_2015_1292_MOESM8_ESM.docx]

**Supplementary Methods**

**Correction for unmeasured systematic variation in the gene expression data**

Principal components (PCs) of the gene expression data matrix were sequentially included as covariates in the expression quantitative trait loci (eQTL) analysis, until 20 PCs were included. The numbers of unique gene expression probes associated with a significant *cis*-eQTL (referred to as *cis*-eQTL probes) were calculated for each at two false discovery rate (FDR) thresholds (0.10 and 0.05). The numbers of significant *cis*-eQTL probes were greatest when the first five PCs of the gene expression data were included as covariates **(Additional file 3)**. Therefore, in addition to the first PC of the genotype data, the first five PCs of the gene expression data were used as covariates in the final eQTL mapping analysis.

**Stepwise association model for the detection of independent *cis*-associations**

For each gene expression probe that had a significant *cis*-eQTL at a particular FDR threshold, eQTL association analysis was re-run, using the genotype of the most significant *cis*-eQTL single nucleotide polymorphism (SNP) as an additional covariate and only testing SNPs that were significant *cis*-eQTL at the particular FDR threshold. Only SNPs with eQTL p-values that were more significant than the p-value corresponding to the FDR threshold in the initial eQTL analysis that was performed without conditioning on the most significant SNP were included. At each iteration, the most significant SNP in the analysis was recorded as an independent *cis*-eQTL and the subsequent iteration of the eQTL analysis was conditioned on its genotype. The analysis was repeated separately for each significant *cis*-eQTL probe until there were no more significant SNPs left after conditioning on the most significant SNP. To account for genes represented by multiple expression probes, SNPs that were associated with the expression level of more than one probe for a single gene were counted only once as independent *cis*-eQTL.

**Identification of *cis*-eQTL associated with colonic diseases and box plots**

The Broad Institute SNP Annotation and Proxy Search (SNAP) tool (<http://www.broadinstitute.org/mpg/snap>) [1] was used to find all SNPs in high linkage disequilibrium (LD) (r^2^ ≥ 0.8) with National Human Genome Research Institute (NHGRI) SNPs associated with colonic diseases (Crohn’s disease [CD], ulcerative colitis [UC] and colorectal cancer [CRC]) within a 500 kilo base (kb) window, using data from HapMap 2 (release 21, release 22), HapMap 3 (release 21) and 1000 Genomes Pilot 1 Utah residents with Northern and Western European ancestry (CEU) as reference. Among these SNPs, those that were significant *cis*-eQTL in the colon with FDR < 0.10 were identified. For each unique target gene that is associated with a disease-associated SNP or one of its LD partners, a box plot of the normalized and log2-transformed gene expression level as a function of SNP genotype was generated **(Figure 2)**. The genotype of the disease-associated SNP was used in the box plot even if it was not significantly associated with the target gene depicted in the box plot (i.e. A SNP that is in high LD with the disease-associated SNP was a significant *cis*-eQTL instead.) If there were more than two probes that were associated with a *cis*-eQTL for the gene, the expression level of the probe showing the most significant association with the disease-associated SNP was used.

**Simulation-based tests for enrichment of functional variants among *cis*-eQTL**

As LD between *cis*-eQTL SNPs could drive the enrichment of functional variants among eQTL, LD had to be accounted for in the enrichment analyses. For each unique *cis*-eQTL target gene (n = 684, FDR < 0.10), one SNP was randomly selected out of all the *cis*-eQTL SNPs that were associated with the expression of that gene. This set of 684 SNPs roughly corresponded to an independent set of *cis*-eQTL SNPs since almost all genes were associated with a single independent *cis*-eQTL SNP (only nine out of 684 significant *cis*-eQTL SNPs were associated with the expression level of more than one gene; see **Table 1**). This sampling procedure was repeated without replacement 1,000 times to generate 1,000 sets of independent *cis*-eQTL. The number of SNPs that were also functional variants (i.e. SNPs in histone marks peaks or population differentiated SNPs [fixation index {F_ST_} > 0.25]) were determined among each set. Next, one of the 1,000 sets of independent *cis*-eQTL SNPs was randomly sampled and used as a template to generate 1,000 matched sets of randomly selected SNPs. Since there was an overrepresentation of higher-frequency variants among the significant *cis*-eQTL compared to all study SNPs **(Additional file 10)**, SNPs were matched on minor allele frequency (MAF) distribution. SNPs were also matched on distance to the nearest transcription start site (TSS) since *cis*-eQTL were more enriched around TSS compared to other SNPs **(Additional file 11)**. First, all SNPs included in the eQTL analysis were partitioned into non-overlapping MAF bins of width 0.05. MAFs of the SNPs in the overall dataset of 48 African American (AA) subjects were used to generate the MAF bins. Within each MAF bin, SNPs were further binned based on their distance to the nearest TSS, creating non-overlapping bins of width 100 kb. Next, 1,000 randomized SNP sets were generated, each of the same size as the set of independent *cis*-eQTL SNPs (n = 684) and matched on MAF distribution and distance to TSS, sampled without replacement from the list of all SNPs included in the eQTL analysis (n = 8,400,922) that had been grouped into discrete MAF and TSS bins. In each of the randomized SNP sets, the number of SNPs that were also functional variants was counted. By comparing the number of functional variants in each of the 1,000 sets of independent *cis*-eQTL to the number of functional variants in each 1,000 randomized SNP set (a total of 1,000 x 1,000 = 10^6^ comparisons), an empirical p-value for enrichment was calculated as the proportion of comparisons in which the number of functional variants in the independent *cis*-eQTL set exceeds that in the randomized SNP set.

**Histone mark data**

Data for the histone mark enrichment analyses came from the Human Genome Atlas (release 9) generated by the National Institutes of Health (NIH) Roadmap Epigenomics Mapping Consortium [2] which uses chromatin immunoprecipitation sequencing (ChIP-Seq) to characterize the genome-wide locations of histone mark modifications in a variety of cell types. Peak tracks for the histone marks were downloaded via the Genboree Workbench (http://[www.genboree.org](http://www.genboree.org)). Enrichment among colon *cis*-eQTL was assessed for four different histone marks associated with gene activation (H3K4me1, H3K4me3, H3K9ac and H3K36me3) and two histone mark associated with gene repression (H3K9me3 and H3K27me3) in two types of colonic cells: mucosa and smooth muscle. Additionally, enrichment of these histone marks was evaluated in three other non-colon tissue types: adult liver, adipose nuclei and breast myoepithelial cells. As some of the histone mark peak tracks were not available for all tissue types on the Genboree website, enrichment could not be tested for all combinations of histone marks and tissue types.

Histone mark enrichment analysis was also conducted in a human colorectal adenocarcinoma cell line (Caco-2). Histone modification data for Caco-2 had been generated as part of the Encyclopedia of DNA Elements (ENCODE) project, which uses ChIP-Seq to provide a detailed map of histone modifications in a variety of cell lines [3, 4]. Histone modification tracks for two activating histone marks (H3K4me3 and H3K36me3) and one repressive histone mark (H3K27me3), referenced to the GRCh37/hg19 Human Genome Assembly, were downloaded from the ENCODE project website (http://encodeproject.org/ENCODE).

**List of abbreviations**

AA: African American

ADHD: Attention deficit hyperactivity disorder

ASW: African ancestry in Southwest USA

BD: Bipolar disorder

bp: Base pair

CD: Crohn’s disease

CEU: Utah residents with Northern and Western European ancestry

ChIP-Seq: Chromatin immunoprecipitation sequencing

*COLCA2*: colorectal cancer associated 2

CRC: Colorectal cancer

ENCODE: Encyclopedia of DNA elements

eQTL: Expression qualitative trait loci

*ERAP2*: Endoplasmic reticulum aminopeptidase 2

FDR: False discovery rate

F_ST_: Fixation index

IBD: Inflammatory bowel disease

kb: Kilo base pair

LD: Linkage disequilibrium

MAF: Minor allele frequency

Mb: Mega base pair

NHGRI: National Human Genome Research Institute

NIH: National Institute of Health

PC: Principal component

PCA: Principal components analysis

RA: Rheumatoid arthritis

*SFMBT1*: Scm-like with four MBT domains 1

SNAP: SNP Annotation and Proxy Search

SNP: Single nucleotide polymorphism

TSS: Transcription start site

UC: Ulcerative colitis

YRI: Yoruba in Ibadan, Nigeria

1. Johnson AD, Handsaker RE, Pulit SL, Nizzari MM, O'Donnell CJ, de Bakker PI: **SNAP: a web-based tool for identification and annotation of proxy SNPs using HapMap**. *Bioinformatics* 2008, **24**(24):2938-2939.

2. Bernstein BE, Stamatoyannopoulos JA, Costello JF, Ren B, Milosavljevic A, Meissner A, Kellis M, Marra MA, Beaudet AL, Ecker JR *et al*: **The NIH Roadmap Epigenomics Mapping Consortium**. *Nature biotechnology* 2010, **28**(10):1045-1048.

3. Consortium EP: **The ENCODE (ENCyclopedia Of DNA Elements) Project**. *Science* 2004, **306**(5696):636-640.

4. Consortium EP, Bernstein BE, Birney E, Dunham I, Green ED, Gunter C, Snyder M: **An integrated encyclopedia of DNA elements in the human genome**. *Nature* 2012, **489**(7414):57-74.

**Supplementary Figure Legends**

**Figure S1.** The 48 African American subjects cluster with HapMap African American samples on the principal components analysis (PCA) plot, indicating mixed African and European ancestry. A PCA-based analysis was performed in EIGENSTRAT to estimate the proportions of European and African ancestry for each subject (represented by PC1). HapMap populations of African ancestry in Southwest USA (ASW), CEU and Yoruba in Ibadan, Nigeria (YRI) were used as references in the PCA plot and are color-coded as indicated in the legend. PC1 was included as a covariate in the eQTL analysis to account for ancestry.

**Figure S2.** Heat map visualization and hierarchical clustering of the gene expression data. Study samples are organized based on the similarity of log2-transformed and quantile-normalized gene expression levels across all probes evaluated in the study. The heat maps represent the pairwise correlation matrix of similarity among the study subjects across all probes. Each row and column represents one of the subjects. The color scale indicates the degree of correlation from lowest (white) to highest (red). (A) Heat map corresponding to the gene expression data for the 48 subjects prior to the removal of outliers. The first eight subjects from the left side of the plot were deemed as outliers and excluded from the eQTL analysis. (B) Heat map corresponding to the gene expression data for the 40 remaining subjects after the removal of outliers.

**Figure S3.** The maximum numbers of gene expression probes associated with a *cis-*eQTL were obtained when the first five PCs from the gene expression data were used as covariates in the eQTL analysis. Each of the first 20 PCs from the gene expression data were sequentially included as covariates in the analysis and the numbers of gene expression probes that were associated with a *cis*-eQTL were determined for each at two different FDR thresholds for SNP-gene associations. The first five PCs were included as covariates in subsequent analyses, to correct for unmeasured variation in the gene expression data.

**Figure S4.** The colon eQTL dataset is enriched for *cis*- but not *trans*-eQTL. (A) The Q-Q plot shows the eQTL association p-values for all SNP-probe pairs (8,400,922 SNPs x 16,252 probes) evaluated in the study. Red dots represent *cis-* and blue dots represent *trans-*association p-values. The gray line is the identity line (y = x) representing the null distribution under which there is no association between SNP genotype and probe expression level. *Cis*-association p-values deviate strongly from the identity line, demonstrating an enrichment of significant associations. There is no such enrichment for *trans*-associations. (B) The histogram shows the eQTL association p-values for all *cis* SNP-probe pairs. There is a clear enrichment of *cis-*associations with small p-values.

**Figure S5.** *Cis*-eQTL cluster roughly symmetrically around TSS. The scatter plot depicts the distribution of *cis*-eQTL relative to TSS. Each dot represents the most significant *cis*-associated SNP for each gene expression probe. –log10(p-value) for the SNP-probe association (y-axis) is plotted against the base pair (bp) distance of the associated SNP from the TSS of the transcript that the probe is interrogating (x-axis). Negative and positive values of the distance denote SNPs 5′ and 3′ of TSS (set at 0), respectively. The majority of significant *cis*-eQTL are found within 100 kb of TSS.

**Figure S6.** Human populations across the world differ in the designations of ancestral and derived alleles as major alleles for UC-associated colon *cis*-eQTL rs9847710. rs9847710 exhibits a relatively high level of population differentiation between 1000 Genomes EUR and AFR populations (F_ST_ > 0.10). The plot shows the distribution of rs9847710 alleles in multiple populations worldwide. The haplotype frequencies for different human populations are depicted in pie charts where the ancestral allele (C) is shown in blue and the derived allele (T) is shown in orange.

**Figure S7.** This is similar to **Figure 4**, except the plots depict the enrichment of colon *cis*-eQTL among SNPs associated with (A) autoimmune disorders, (B) cancers and (C) psychiatric disorders. There is no significant enrichment for colon *cis*-eQTL among SNPs associated with these disorders.

**Figure S9.** This is similar to **Figure 5**, except the histone mark data is from a colorectal adenocarcinoma cell line (Caco-2) and only two active histone marks (H3K4me3 and H3K36me3) and one repressive histone mark (H3K27me3) are depicted. Colon *cis*-eQTL are enriched for active histone marks with the enrichment reaching statistical significance for H3K4me3 (p < 1.0 x 10^-6^) and being highly suggestive for H3K36me3 (p = 0.060). There is no statistically significant enrichment of the repressive histone mark H3K27me3.

**Figure S10.** MAF distribution of significant *cis*-eQTL is skewed towards higher frequencies compared with the complete set of study SNPs. MAF distribution of the complete set of study SNPs (top graph) is presented, along with the MAF distributions of *cis*-eQTL at four different FDR thresholds. The skew towards higher frequencies becomes more pronounced as the FDR threshold becomes more stringent.

**Figure S11.** *Cis*-eQTL are closer to TSS than other SNPs. The histograms show the distributions of the base pair (bp) distances to the nearest TSS for all study SNPs that are within 1 mega base pair (Mb) of a TSS and significant *cis*-eQTL at various FDR thresholds. Negative distances refer to SNPs upstream (5’) of the TSS (set at 0) while positive distances refer to those downstream (3’).

**Supplementary Tables**

| **Table S1. Enrichment of colon *cis*-eQTL among disease-associated SNPs** | | | |
| --- | --- | --- | --- |
|  | **Number of SNPs** | |  |
|  | **Disease-associated SNPs** | **Disease-associated SNPs that are colon *cis*-eQTL** | **p-value*** |
| **Lipid traits** | 210 | 5 | 0.203 |
| **Celiac disease** | 57 | 0 | 0.659 |
| **Psoriasis** | 35 | 0 | 0.504 |
| **RA** | 54 | 1 | 0.253 |
| **Breast cancer** | 100 | 0 | 0.575 |
| **Melanoma** | 16 | 0 | 0.250 |
| **Prostate cancer** | 121 | 1 | 0.711 |
| **ADHD** | 179 | 5 | 0.117 |
| **BD** | 257 | 3 | 0.728 |
| **Schizophrenia** | 247 | 6 | 0.214 |

*P-values were empirically determined using a simulation-based method in which 1,000 randomized SNP sets, matched in size and MAF distribution to the disease-associated SNPs, were generated. These simulations yielded a p-value, calculated as the proportion of sampled SNP sets in which the *cis*-eQTL count exceeds the actual count observed in the disease-associated SNPs. For details see **Supplementary Methods**.
